# Progesterone receptor antagonists reverse stem cell expansion and the paracrine effectors of progesterone action in the mouse mammary gland

**DOI:** 10.1186/s13058-021-01455-2

**Published:** 2021-08-03

**Authors:** Manish Ranjan, Oukseub Lee, Gannon Cottone, Elnaz Mirzaei Mehrabad, Benjamin T. Spike, Zexian Zeng, Shivangi Yadav, Robert Chatterton, J. Julie Kim, Susan E. Clare, Seema A. Khan

**Affiliations:** 1 Department of Surgery, Feinberg School of Medicine, Northwestern University, Chicago, IL 60611 USA; 2 School of Computing, University of Utah, Salt Lake City, UT 84112 USA; 3 Huntsman Cancer Institute, Department of Oncological Sciences, University of Utah, Salt Lake City, UT 84112 USA; 4 Division of Health and Biomedical Informatics, Department of Preventive Medicine, Feinberg School of Medicine, Northwestern University, Chicago, IL 60611 USA; 5 Department of Obstetrics and Gynecology, Feinberg School of Medicine, Northwestern University, Chicago, IL 60611 USA; 6 Robert H. Lurie Comprehensive Cancer Center of Northwestern University, Chicago, IL 60611 USA

**Keywords:** Mammary stem cells, Luminal progenitor cells, estrogen and progesterone, Progesterone receptor modulators, Epithelial-mesenchymal transition, Alternative RNA splicing

## Abstract

**Background:**

The ovarian hormones estrogen and progesterone (EP) are implicated in breast cancer causation. A specific consequence of progesterone exposure is the expansion of the mammary stem cell (MSC) and luminal progenitor (LP) compartments. We hypothesized that this effect, and its molecular facilitators, could be abrogated by progesterone receptor (PR) antagonists administered in a mouse model.

**Methods:**

Ovariectomized FVB mice were randomized to 14 days of treatment: sham, EP, EP + telapristone (EP + TPA), EP + mifepristone (EP + MFP). Mice were then sacrificed, mammary glands harvested, and mammary epithelial cell lineages separated by flow cytometry using cell surface markers. RNA from each lineage was sequenced and differential gene expression was analyzed using DESeq. Quantitative PCR was performed to confirm the candidate genes discovered in RNA seq. ANOVA with Tukey post hoc analysis was performed to compare relative expression. Alternative splicing events were examined using the rMATs multivariate analysis tool.

**Results:**

Significant increases in the MSC and luminal mature (LM) cell fractions were observed following EP treatment compared to control (*p* < 0.01 and *p* < 0.05, respectively), whereas the LP fraction was significantly reduced (*p* < 0.05). These hormone-induced effects were reversed upon exposure to TPA and MFP (*p* < 0.01 for both). Gene Ontology analysis of RNA-sequencing data showed EP-induced enrichment of several pathways, with the largest effect on *Wnt* signaling in MSC, significantly repressed by PR inhibitors. In LP cells, significant induction of *Wnt4* and *Rankl*, and *Wnt* pathway intermediates *Lrp2* and *Axin2* (confirmed by qRTPCR) were reversed by TPA and MFP (*p* < 0.0001). Downstream signaling intermediates of these pathways *(Lrp5*, *Mmp7*) showed similar effects. Expression of markers of epithelial-mesenchymal transition (*Cdh1*, *Cdh3*) and the induction of EMT regulators (*Zeb1*, *Zeb2*, *Gli3*, *Snai1*, and *Ptch2*) were significantly responsive to progesterone. EP treatment was associated with large-scale alternative splicing events, with an enrichment of motifs associated with *Srsf*, *Esrp*, and *Rbfox* families. Exon skipping was observed in *Cdh1*, *Enah*, and *Brd4*.

**Conclusions:**

PR inhibition reverses known tumorigenic pathways in the mammary gland and suppresses a previously unknown effect of progesterone on RNA splicing events. In total, our results strengthen the case for reconsideration of PR inhibitors for breast cancer prevention.

**Supplementary Information:**

The online version contains supplementary material available at 10.1186/s13058-021-01455-2.

## Background

Over the past decade, the role of the ovarian steroid hormone progesterone in the development of breast cancer has been increasingly recognized, supported by both murine models and human data [1]. One dimension of this cancer-promoting effect relates to the finding that progesterone, in the presence of estrogen, generates a significant increase in the number of murine mammary stem cells (MSC) and influences the intrinsic ability of these cells to regenerate the mammary gland [2, 3]. MSC, located in a specialized niche in the basal epithelium of the mouse mammary gland [4], are capable of giving rise to basal and luminal cells [5–7]. This self-renewing cell population increases several fold in the presence of steroid hormones despite a lack of estrogen receptor (ER) or progesterone receptor (PR) expression. This expansion has been shown to result from the paracrine effects of receptor activator of nuclear factor kappa-B ligand (*Rankl*) and *Wnt4* produced in the luminal compartment [3, 8], which is comprised of luminal mature (LM) and luminal progenitor (LP) cells.

The increase in MSC numbers in response to the ovarian steroid hormones, demonstrated in the mouse data derived by Asselin-Labat [2] and Joshi [3], contributes to a heightened breast cancer risk, since increased stem cell divisions will promote the accumulation of replicative mutations that facilitate oncogenesis [9, 10]. Using ovariectomized mice treated with exogenous hormones, Asselin-Labat and colleagues [2] observed a transient 11-fold increase in MSC at mid-pregnancy, when serum levels of progesterone are at their highest. Joshi et al. studied the effects of endogenous EP exposure and noted an almost 2-fold increase in MSC at diestrus that translated into a 14-fold increase in the absolute number of mammary repopulating units [3], with increased numbers of both MSCs and LMs. In a subsequent study using a different cell sorting strategy, these investigators demonstrated that exposure to EP in addition to increasing the number of MSC, increases the number of ER/PR negative luminal progenitor cells [8].

Human studies also implicate combined exposure to EP in the promotion of breast cancer. Risk increases with a higher number of ovulatory cycles [9, 10], and with the inclusion of progestins in postmenopausal hormone therapy [11, 12].

Given this abundance of preclinical and clinical data, the recent availability of relatively potent progesterone antagonists with minimal antiglucocorticoid activity, such as telapristone acetate (TPA) and ulipristal acetate (UPA), has prompted renewed interest in selective PR modulators (SPRMs) for breast cancer prevention and therapy [11–17]. Therefore, we have used a murine model to determine the effects of EP ± PR blockade with either MFP or TPA on the epithelial cell compartments of the mouse mammary gland. We assessed whether PR blockade (with or without a GR component, as seen with mifepristone) affects the distribution of cell lineages and gene expression within the compartments, and whether the EP-driven increase in the MSC compartment is prevented by this blockade. We then performed RNA sequencing on specific mammary lineages, to evaluate molecular pathways associated with the observed compartmental effects. We chose not to include single-hormone comparison groups in our experiment (E alone and P alone) since, from a translational perspective, endogenous estrogen exposure can occur alone, or with progesterone, but progesterone exposure is always accompanied by some estrogen exposure. In addition, in vitro studies have revealed that when both hormones are present progesterone modulates estrogen’s actions to the extent that they resemble those of progesterone alone [18].

Progesterone’s tumorigenic effects likely extend beyond the number of stem cell divisions. Among the pathways identified in our RNA-sequencing data to be significantly induced by EP, which was reversed by EP + PR blockade, is the epithelial to mesenchymal transition (EMT), a sequential process of cells transitioning from an immotile polarized state to a migratory mesenchymal-like state [19, 20]. MSCs depend on EMT programs to support their stemness [21, 22]. Progesterone exposure stimulates WNT4/β-catenin signaling, which enables an EMT permissive state through E-cadherin downregulation [23, 24]. Similarly, progestin-based hormonal contraceptives induce WNT4/β-catenin signaling and activate ZEB1 in human mammary tissue [24–26]. ZEB1 is a transcription factor which facilitates EMT resulting in cellular migration and invasion [27]. A reanalysis of PR ChiP-seq data derived from mammary epithelial cells revealed that PR binding sites are present in the upstream promoter region of the EMT regulating transcription factors ZEB1, ZEB2, and SNAI1 [28]. Close coordination of feedback among chromatin topology, transcription, and alternative splicing regulate EMT-associated gene expression [29]. We, therefore queried our RNA-seq data to gain insights into how EP and the blockade of PR affect EMT, a cancer hallmark-facilitating program [30].

## Methods

A schematic of the experimental workflow of is presented in Supplementary Fig. 1.

### Mouse strain, animal surgery, and treatment:

All animal experiments were approved by the IACUC at Northwestern University. Eight-week-old female ovariectomized FVB (Jackson Laboratories, Bar Harbor, ME) mice weighing > 20 g, were randomized into 4 treatment groups (nine/group): sham (skin incision only, no pellets), EP, EP + Telapristone Acetate (TPA), EP + Mifepristone (MFP). At age 10 weeks, hormones and drugs were subcutaneously implanted between neck and shoulder as a 30-day release pellet containing EP (0.3 mg of 17 β-estradiol and 30 mg of progesterone), TPA (30 mg), and MFP (30 mg). The pellets were custom-ordered from the Innovative Research of America (Sarasota, FL). TPA was a gift from Repros Therapeutics Inc. (Woodlands, TX). On day 15 of treatment, the mice were sacrificed to collect mammary glands.

### Mammary cell isolation:

Freshly harvested 4th inguinal mammary gland pairs were weighed, minced, and digested with collagenase solution (1 ml/0.1gram of tissue) in a 15-ml Falcon tube, then shaken at 37 °C for 1.5 h. The solution contained 1:4 collagenase (Stemcell Technologies, Vancouver, BC, Canada) in DMEM-F12 (Gibco, Carlsbad, CA) supplemented with 5% FBS (Gibco, Carlsbad, CA). Next, the samples were treated with red blood cell lysis buffer (Stemcell Technologies, Vancouver, BC, Canada) and a single cell suspension was prepared by treatment with 0.05% trypsin for 5 min followed by 10-min incubation in Dispase with DNAse I (Stemcell Technologies, Vancouver, BC, Canada). Lastly, the suspension was filtered through a 40-μm mesh and aliquoted into 1.5-ml Eppendorf tubes for antibody labeling.

### Antibodies:

All antibodies raised against mouse antigen: CD-45 (30-F11), TER-119 (TER-119), CD-31 (390), CD-24 (M1/69), CD-49f (GOH3), were obtained from Ebioscience (San Diego, CA) and CD-61 (2C9.G2/HMβ3-1) was obtained from BDBiosciences (Franklin Lakes, New Jersey).

### Flow-assisted cell sorting:

**Fig. 1:**
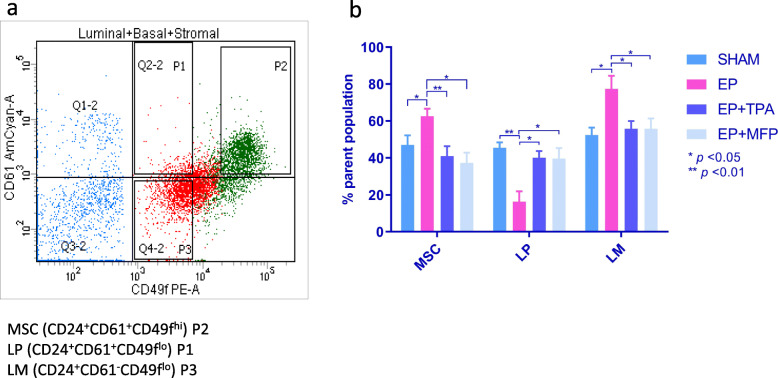
The response of mammary epithelial cell lineages to E + P (estrogen + progesterone) exposure is reversed by PR blockade. **a** Representative separation of cellular lineages by FACS. **b** Cell expansion was significantly attenuated in both TPA- (*p* < 0.010) and MFP- (*p* < 0.01) treated mice compared to MaSC in mice treated with EP alone. The LP cells show marked reduction in EP-treated mice (*p* < 0.01) compared to sham. LM cells show an expansion (*p* < 0.05) in EP-treated mice compared to sham (52.40%). TPA significantly (*p* < 0.01) suppresses LM cell expansion observed in the EP group. LP cells show significant expansion in both EP + TPA (*p* < 0.01) and EP + MFP (*p* < 0.05) group. The MaSc within the basal cell niche show significant expansion at day 15 when mice were sacrificed (*p* < 0.05) in mice implanted with E2 and P4 compared to sham. The relative percentage of cells stained were compared by one-way ANOVA and Tukey post hoc analysis to compare between specific groups. Parental cells are the CD24+ cells

As described by Joshi [3], lineage-negative mammary gland cells were sorted into luminal and basal population subsets (Fig. 1a). A flow diagram of the cell sorting strategy is presented as Supplementary Fig. 2. After isolating lineage-negative cells, CD24 and CD49 were employed to define the basal epithelial population (CD24^+^CD49f^hi^) and the luminal population (CD24^+^CD49f^low^). Using CD61, cell lineages were further defined into MSCs (CD61^+^CD24^+^CD49f^hi^), LP cells (CD61^+^ CD24^+^CD49f^lo^), and LM cells (CD61^−^ CD24^+^CD49f^lo^). Cell populations were double sorted to enhance purity on BD FACSAria 5-Laser (Franklin Lakes, NJ) and analyzed using BD FACSDIVA II (Franklin Lakes, NJ). The percentages of cells stained were compared using one-way ANOVA and Tukey’s post hoc analysis to compare between specific treatments. The cells were directly sorted into the lysis buffer, briefly vortexed and snap frozen for RNA extraction.

### RNA isolation and library preparation

Qiagen RNEasy miniprep kit (Germantown, MD) was used to isolate RNA from the cells sorted directly into the lysis buffer, briefly vortexed and snap frozen. The concentration and quality of total RNA in samples was assessed using Agilent 2100 Bioanalyzer (Santa Clara, CA). One nanogram of RNA/sample with RNA Integrity Number ≥ 5 was used to prepare a dual-indexed cDNA library using SMARTer RNA Pico Kit v2 (Clontech, Mountain View, CA). The resulting libraries were assessed for quantity and size distribution using Qubit (Thermo Fisher, Waltham, MA) and the Agilent 2100 Bioanalyzer (Santa Clara, CA). In total, 200 pM pooled libraries were utilized per flowcell for clustering amplification on cBot using HiSeq 3000/4000 PE Cluster Kit (Illumina, San Diego, CA) and sequenced with 2 × 75 bp paired-end configuration on HiSeq4000 (Illumina) using HiSeq 3000/4000 PE SBS Kit (Illumina) at the Center for Medical Genomics at Indiana University. A Phred quality score (Q score) was used to measure the quality of sequencing. The sequencing reads reached Q30 in > 90% of samples (99.9% base call accuracy).

### Processing and analysis of RNA-seq data

Reads were aligned to the mouse reference genome GRCm38 primary assembly sourced from GENCODE [31] project using STAR (ver 2.6) [32]. Htseq-count [33] (ver 0.5.4) was used to quantitate the gene expression level where a gene was considered as the union of all its exons. For gene annotation, GTF file was sourced from GENCODE [31]. For differential gene expression analyses, EdgeR [34, 35]/DESeq2 [36] was used. Data was first filtered for low counts and normalized for sequencing depth, gene length, and library size before fitting a negative binomial model. Fisher’s exact test was performed using the tagwise dispersion approach. Pathway enrichment was performed using the gene-set enrichment analysis [37].

The differential gene expression results were verified by having the data analyzed on the Artificial Intelligence platform (Intelligene Technologies, Kenosha, WI). Short reads were aligned to the mm10 mouse genome using STAR [32]. Subsequently cufflink packages were used to perform transcript assemblies [38]. Downstream differential gene expression calling on the reference and experimental groups of interest was performed using DESeq2 [36]. To perform clustering analyses on a group of samples, a union of all the genes and their expression RPKM values within that group was generated to build a read-count matrix for the group of interest. Various unsupervised and other machine learning techniques were applied to this composite read-count matrix of interest. The sample-feature heatmaps represent the signal intensity of a feature for any given sample. Functional analysis was performed using gene-set enrichment analyses [37].

### Pathway enrichment

The online pathway enrichment tool David [39] was used to examine the GO term enrichment in MSC, LP, and LM cells.

### Validation of candidate genes from RNA-seq

To validate the candidate genes identified in our high-throughput transcriptomic study, total RNA from the inguinal mammary gland was reverse transcribed to cDNA (Biorad iScript cDNA synthesis kit, Hercules, CA), and amplified using quantitative PCR (qRTPCR). Primer sequences for qRTPCR were obtained from Primer bank (Supplementary Table 1) [40–42], and primers were synthesized by IDT DNA (Coralville, IA). PowerUP SYBR from Applied Biosystems (Foster City, CA) was used to amplify the target genes. Livak’s fold change [43] was estimated relative to 18S RNA as the house-keeping gene. ANOVA with Tukey post hoc analysis was performed to compare the relative expression between different groups.

### Analysis of alternative splicing

Multivariate analysis of transcript splicing was performed using rMATS package (v4.0.2) [44–46]. Reads were aligned to mouse reference genome GRCm38 primary assembly sourced from the GENCODE [31] project using STAR (ver 2.6) [32]. STAR aligner performs soft clipping by default which generates variable read lengths. Since rMATS [44–46] requires that all of the reads are the same length, we use “--alignEndsType EndToEnd” parameter to suppress default soft clipping of reads performed by STAR. rMATS [44–46] was run on the aligned BAM files. For gene annotation, GTF file was sourced from GENCODE [31]. Choosing an FDR of < 0.1%, significant splicing events were determined. To further understand the RNA-binding proteins and splicing factors, the motifs adjacent to splice sites were examined using RNA maps [47]. Motifs enriched for RNA-binding proteins and splicing factors were determined in MSC, LP, and LM cell types.

### Validation of alternative splicing using scRNA-sequencing data sets

A flow diagram of the splicing analysis workflow is presented in Supplementary Fig. 3. FASTQ files from Bach et al. [48] were downloaded from the Gene Expression Omnibus (GEO) at https://www.ncbi.nlm.nih.gov/geo/query/acc.cgi?acc = GSE106273) and Cell Ranger at https://support.10xgenomics.com/single-cell-gene-expression/software/pipelines/latest/what-is-cell-ranger utilized to generate .bam and .cloupe files. Subsequently, cell transcriptomes were clustered independently for each replicate and developmental stage described in Bach et al. to delineate major cell groups using k-means clustering as embedded in Loupe Browser [48]. For each resulting cluster, we tabulated the mean expression of Krt18 and Krt5 and the proportion of positive cells. Based on these data, cells were designated as belonging to Krt5-high, Krt18-high, Krt18-low, and “other” clusters (Supplementary Fig. 4). The Subset-bam tool (https://github.com/10XGenomics/subset-bam) was then used to generate independent .bam files for each cluster type based on the cell name/barcode extracted from Loupe Browser clustering. Krt5-high and Krt18-high clusters representing basal (BC) and luminal cells (LC), respectively, were subsequently analyzed for alternative splicing (Supplementary Figure 4). The resulting cell type, LC or BC, and stage-specific, Nulliparous (NP) or Gestational (G), .bam files were then unaligned with Samtools (http://www.htslib.org/doc/samtools.html) and re-mapped to GRCm38 (https://www.gencodegenes.org/mouse/release_M25.html) using STAR [32] to generate SJ.out.tab files containing splice junction reads for analysis with Outrigger [49].

The data from rMATS LP/MSC: CvEP, CvEPT, and EPvEPT were filtered for events meeting statistical significance (*p* value < 0.05, FDR < 0.1). After filtering for statistical significance, the events from each treatment comparison were again filtered so only events unique to LP CvEP and MSC CvEP remain. The significant and unique AS events were then compared to the Krt18-high (luminal) and Krt5-high (basal) Outrigger results from each stage based on genomic coordinates (with a buffer ± 20 base pairs).

## Results

### The response of mammary epithelial cell lineages to EP exposure is reversed by PR blockade

We first examined epithelial cell number and fraction in each lineage (MSC, LP, LM) based on flow cytometry (Fig. 1a) and confirmed the lineage of the isolated cells by quantitative real-time polymerase chain reaction (qRTPCR). MSC percent was significantly greater in the EP group compared to sham (*p* < 0.05), and this effect was reversed upon exposure to PR inhibitors TPA and MFP (*p* < 0.01) (Fig. 1b). Within the luminal subpopulations, the LP cells showed a significantly reduced cell fraction upon EP exposure (*p* < 0.01) and LM cells showed a significant expansion after EP treatment (*p* < 0.05) compared to sham. The EP-mediated expansion of both LM and MSC subpopulations was reversed upon exposure to TPA and MFP (*p* < 0.01) (Fig. 1b), which also reversed the EP-mediated decrease of LP cells; the magnitude of the effect was very similar for both agents. The lineage of the isolated cells was confirmed by qRTPCR (Supplementary Figure 1b-d). *Krt14* (Supplementary Figure 1b) and *Acta2*/*Sma* (Supplementary Figure 1c) showed higher expression in the MSC cells than in LP and LM cells, and *Gata3* (Supplementary Figure 1d) and *Krt18* (Supplementary Figure. 1e) showed higher expression in luminal populations; the PR antagonists had no effect on these lineage markers.

### TPA and MFP block EP-induced paracrine effectors in MSC and LM cell populations

**Fig. 2:**
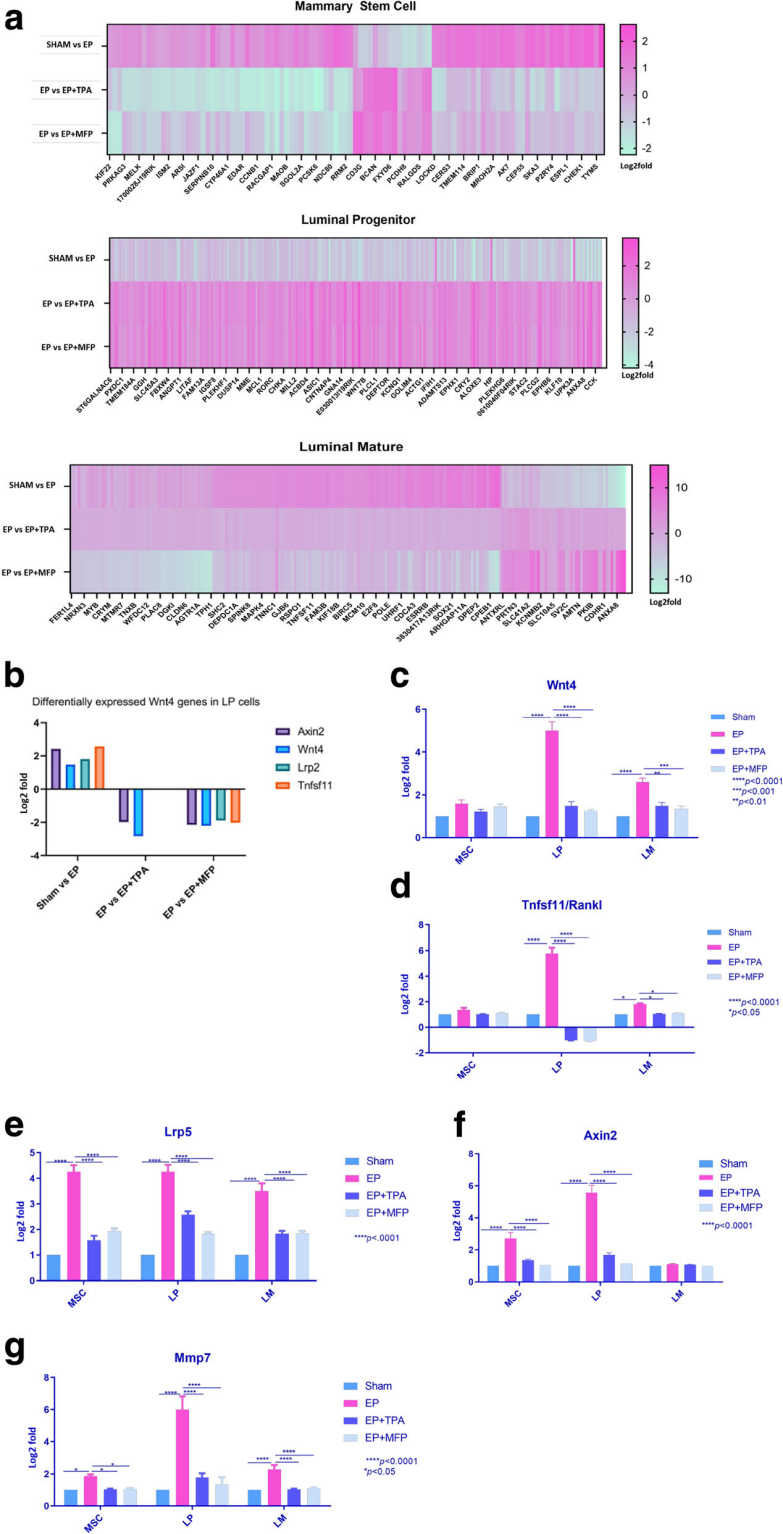
Progesterone receptor mediated induction of the WNT/RANKL-driven mitotic signal in mammary epithelial cells. **a** Heat map showing differentially expressed genes (adjusted *p* value < 0.05) between Sham and EP and EP vs EP + PR inhibitor in MaSC, LP, and LM cells. **b** In RNA-seq data, LP cells show highest induction of *Wnt4*/*Rankl-*driven signal in EP-treated group. **c**, **d** qRTPCR was performed to confirm the downstream signaling of *Wnt4*/*Rankl* across all cell lineages. Livak’s fold change was calculated relative to MaSC (Sham). **c** A significant induction of *Wnt4* expression was observed in LP and LM cells upon EP exposure relative to Sham (*p* < 0.0001); this effect was not observed in mice treated with EP + PR inhibitors, LP (*p* < 0.0001), LM EP + TPA (*p* < 0.01), and LM EP + MFP (*p* < 0.001). **d** LP cells in EP-treated mice show a strong induction of *Rankl*/*Tnfsf11* (*p* < 0.0001). This induction was not observed in mice group treated with EP + PR inhibitors (*p* < 0.0001). LM cells in EP-treated mice also show significant albeit lesser induction of *Rankl*/*Tnfsf11* (*p* < 0.05) and was reversed in EP + PR inhibitor-treated mice (*p* < 0.05). The MaSC compartment showed a non-significant induction of *Rankl*/*Tnfsf11* signaling (*p* > 0.05). **e** *Lrp5* expression was significantly induced in all three lineages after treatment with EP relative to sham (*p* < 0.0001) and suppressed in EP + PR inhibitor-treated mice (*p* < 0.0001). **f** *Axin2* mRNA expression is significantly induced (*p* < 0.001) in EP-treated group in MSC and LP cells relative to sham, which is abrogated in mice treated with EP + PR inhibitors (*p* < 0.0001). **g** *Mmp7* mRNA expression was induced both LP and LM cells in EP-treated mice compared to sham group (*p* < 0.0001), and this effect was reversed in EP + PR inhibitor-treated mice (*p* < 0.0001). MaSc cells show a significant induction of *Mmp7* expression (*p* < 0.05) in EP group relative to sham group and this effect is reversed in mice treated with EP + PR inhibitors (*p* < 0.05)

Evaluation of RNA-seq data revealed genes that were differentially expressed in control versus EP and reversed in the EP + TPA and EP + MFP groups in MSC, LP, and LM lineages; these are presented in Fig. 2a (The number of genes displayed in the heat maps has been limited in order to enhance legibility. Complete lists of differentially expressed genes are provided in Supplementary Tables 2, 3 and 4.) Across all three lineages, no differential gene expression was observed between mice implanted with EP + TPA and mice implanted with EP + MFP. The enrichment for Gene Ontology (GO) terms was examined using the online tool DAVID [39]. Enrichment of the *PI3K* pathway, cell cycle proteins, and *Wnt* signaling was observed in all three cell populations in response to EP and repression of the same after treatment with EP + PR inhibitors (Supplementary Fig. 5a-c; Supplementary Tables 2, 3 and 4 color coding). FAS receptor signaling was enriched uniquely in the LP cells.

MSC are ER/PR negative, and thus, it is assumed that progesterone activates paracrine signaling in the luminal and/or the stromal compartments, which then initiates the mitotic signal in the MSC niche. Progesterone-induced MSC expansion is known to be mediated by *Wnt*/*Rankl* signaling [3, 8]; our RNA-seq data showed a small, non-significant EP-induced induction of *Wnt4* and *Rank*l expression in MSC cells, but this effect was strong in LP cells, along with that of the *Wnt* pathway intermediates *Lrp2* and *Axin2*. Importantly, these effects are reversed by TPA (Fig. 2a, b; Supplementary Tables 2, 3 and 4). These RNA-seq findings were confirmed by qRTPCR, with induction of *Wnt4* (Fig. 2c) in LP cells by EP, and reversal upon treatment with MFP and TPA (*p* < 0.0001), thereby indicating mediation by PR. In LP, *Rankl*/*Tnfsf11* (Fig. 2d) expression was increased approximately fivefold by EP treatment (*p* < 0.0001), and again repressed by treatment with MFP (*p* < 0.0001) and TPA (*p* < 0.0001). LM cells also showed a significant, albeit more modest induction of *Wnt4* (*p* < 0.0001) and *Rankl* (*p* < 0.01) by EP, reversed by treatment with PR inhibitors.

We validated the effects on additional downstream signaling intermediates in these pathways vis-à-vis *Lrp5* (Fig. 2e), *Axin2* (Fig. 2f), and *Mmp7* (Fig. 2g); LRP5 is a Wnt receptor which once activated, stabilizes cytosolic β-catenin by preventing its phosphorylation [50]. The expression of *Lrp5* was consistently increased by EP treatment across all three cell lineages (*p* < 0.0001) and reversed by PR inhibitors. AXIN2 is a protein that is recruited to the intracellular domain of the LRP5/6 trimer to enable β-catenin stabilization [50]. We observed a significant induction of *Axin2* mRNA expression (*p* < 0.001) after EP treatment in MSC and LP, which was reversed upon exposure to MFP and TPA (*p* < 0.0001). *Mmp7* (Matrix metalloprotease 7) is a member of Mmp family. It is a transcriptional target of *Wnt*/*Ctnnb1* pathway. Confirmatory qRTPCR in each cell lineage after exposure to EP showed significantly higher expression of *Mmp7* in both LP and LM cells (*p* < 0.0001), again reversed by MFP and TPA (*p* < 0.0001), indicating mediation by PR. MaSc cells in the basal compartment also showed a significantly higher expression of *Mmp7* (*p* < 0.05) in EP groups relative to sham, and its reversal in EP + MFP and EP + TPA groups (*p* < 0.05 in both). Thus, we observed the expected EP induction of paracrine signaling in the LP (CD24^+^CD49f^lo^CD61^−^) subpopulation, with remarkably consistent reversal of these effects to the levels seen in controls, with the addition of either TPA or MFP.

### EP treatment induced epithelial to mesenchymal transition-like profile

**Fig. 3:**
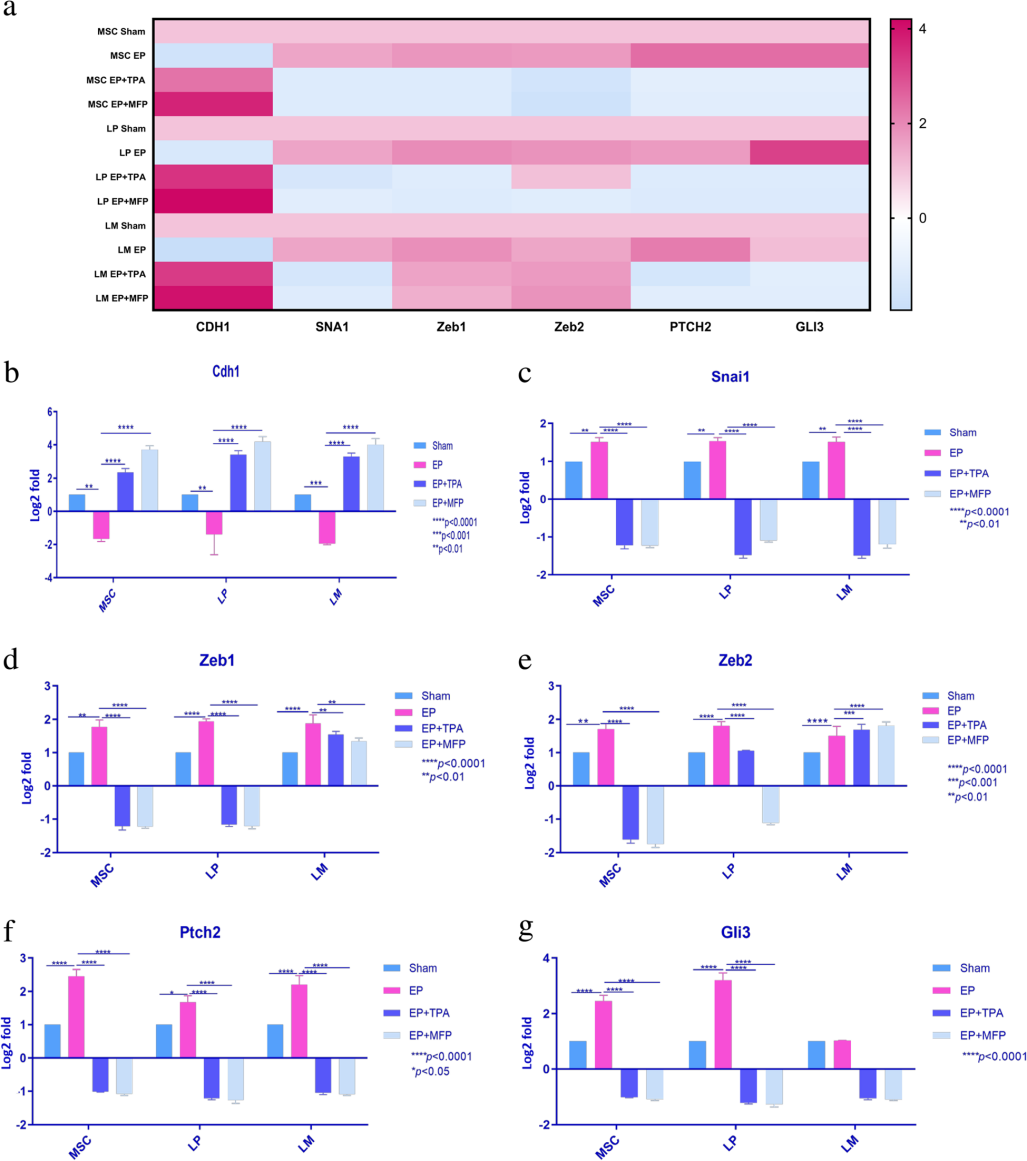
PR regulated induction of EMT-associated genes in the mouse mammary gland. **a** MaSc, LP, and LM cells were examined for the induction of EMT-associated genes in RNA seq data. **b–g** qRTPCR was performed to confirm the mRNA expression and Livak’s fold change was calculated relative to MaSC (Sham). **b** *Cdh1* expression was significantly repressed in EP-treated MaSC (*p* < 0.01), LP (*p* < 0.01), and LM (*p* < 0.001) subpopulations, which was reversed by treatment with EP + PR inhibitors (*p* < 0.0001). **c** *Snai1* showed significantly increased expression in all three subpopulations (*p* < 0.01) in EP-treated group relative to sham and significantly decreased expression in mice treated with EP+ PR inhibitors (*p* < 0.001). **d** *Zeb1* was induced in the EP-treated mice relative to sham-treated mice in the MaSC (*p* < 0.01) and LP and LM compartments (*p* < 0.0001). EP+ PR inhibitor-treated mice show a repression of this induction in both luminal (LP, *p* < 0.0001; LM, *p* < 0.01) and basal compartments (*p* < 0.0001). **e** *Zeb2* was also significantly induced upon exposure to EP in MSC (*p* < 0.001), LP (*p* < 0.0001), and LM (*p* < 0.0001) relative to sham. Mice treated with EP + PR inhibitors showed a significantly lower expression of *Zeb2* relative to EP in MSC and LP cells. LM cells showed an induction of *Zeb2* expression in EP + TPA (*p* < 0.001) and EP + MFP mice (*p* < 0.0001). **f** *Ptch2* shows a significant induction in the EP-treated mice compared to the sham group in all the three subpopulations LP (*p* < 0.05), LM (*p* < 0.0001), and MaSC (*p* < 0.0001). Treatment with EP + PR inhibitors shows a reversal of this effect with significant downregulation observed in all three lineages (*p* < 0.0001). **g** *Gli3* expression in the EP group relative to sham also show an upregulation in MaSc (*p* < 0.0001) and LP (*p* < 0.0001) cells but not in LM cells. The induction of this effect was reversed after treatment with EP + MFP (*p* < 0.0001) and EP + TPA (*p* < 0.0001)

Our RNA-sequencing data in the mammary epithelial cell lineages shows enrichment of cell-cell adhesion molecules (Supplementary Figure 5); disruption of cell-cell adhesion is a key event in the induction of EMT [51, 52]. This association prompted us to further focus our investigation on EMT (Fig. 3a, Supplementary Figure 6). Induction of key EMT markers upon EP treatment was confirmed using qRTPCR. *Cdh1* (Fig. 3b) was significantly repressed after EP treatment in the MSC (*p* < 0.01), LP (*p* < 0.01) and LM (*p* < 0.001) subpopulations and a reversal of these effects was observed with exposure to TPA (*p* < 0.0001) and MFP (*p* < 0.0001) in each of the three subpopulations. qRTPCR confirmed the alteration of expression of the EP-induced transcription factors that repress expression of E-cadherin [53–55]. *Snai1* (Fig. 3c) showed a significantly greater expression in both luminal (*p* < 0.01) and MSC (*p* < 0.01) populations of the EP-treated mice. Treatment with MFP and TPA reversed this effect inducing a significant downregulation (*p* < 0.001) of *Snai1* expression. *Zeb1* (Fig. 3d) and *Zeb2* (Fig. 3e) were both induced in the EP treatment group in MSC cells (*p* < 0.01), as well as in LP and LM compartments (LP *p* < 0.0001, LM *p* < 0.0001); repression of this induction was seen in both LP (*p* < 0.0001) and MSC compartments (*p* < 0.0001). In LM cells, this repression was attenuated (*Zeb1*) or non-existent (*Zeb2*). Induction of EMT involves Hedgehog pathway upregulation [23, 24]. Hedgehog ligands induce Patched-2 (*Ptch2*), which can relieve repression of Smoothened (*Smo*), permitting the expression of *GlI* transcription factors [23, 24]. qRTPCR was performed to examine the expression of *Ptch2* (Fig. 3f) and *Gli3* (Fig. 3g). *Ptch2* showed a significant induction in the EP-treated group compared to the sham in all three lineages. This effect was reversed after treatment with MFP (*p* < 0.0001) and TPA (*p* < 0.0001). qRTPCR performed to examine expression of *Gli3* showed a significantly high induction of *GlI3* expression in MSC (*p* < 0.0001) and LP (*p* < 0.0001) population but not in the LM population, repressed in both by treatment with MFP (*p* < 0.001) and TPA (*p* < 0.001). These results show a synchronous induction of genes linked to an EMT-like profile with EP treatment in MSC and LP, which is prevented by the PR inhibitors, and therefore suggests it is progesterone-mediated.

### P4 exposure induces alternate isoform expression in mammary epithelial cell lineages

**Fig. 4:**
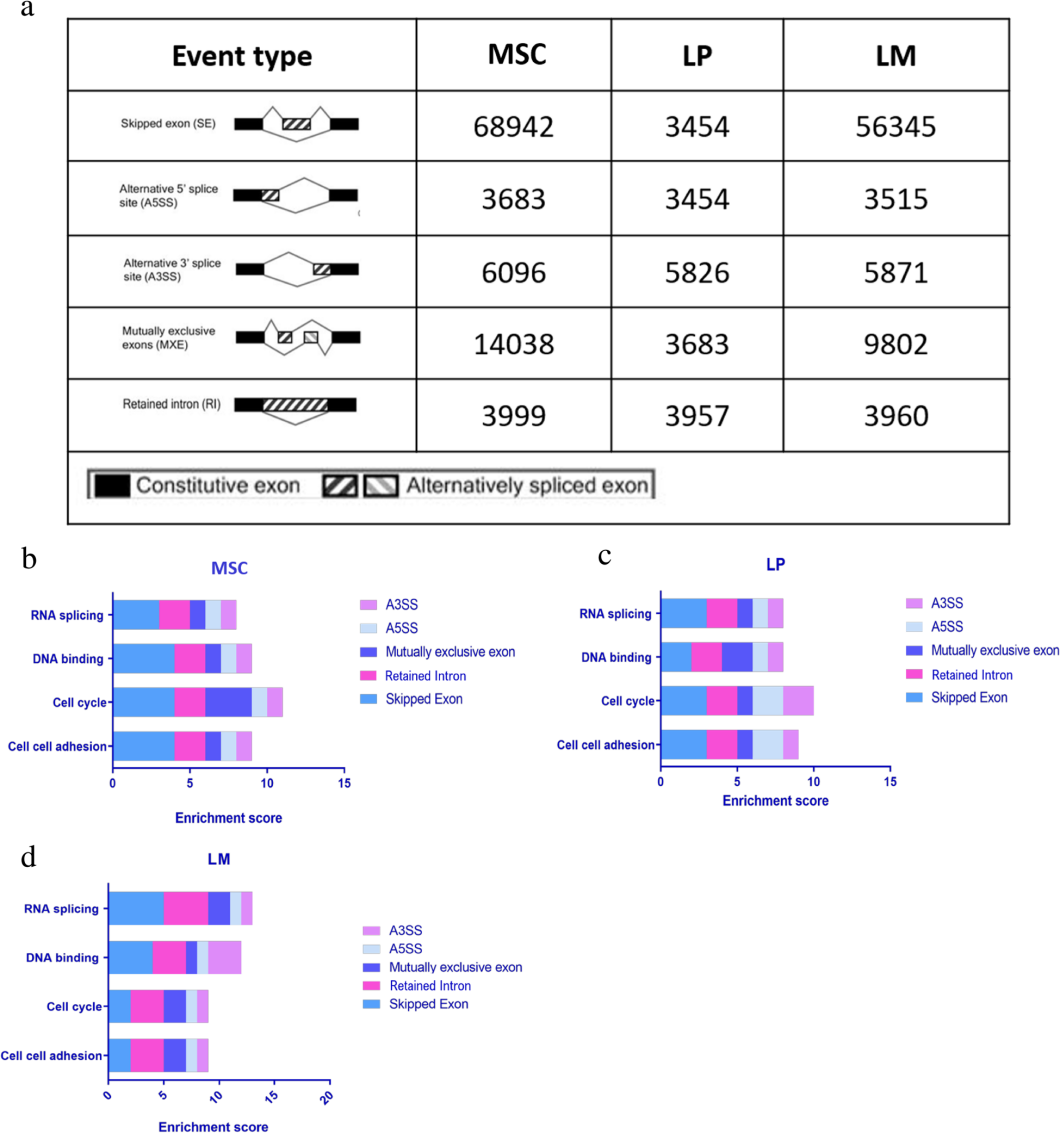
Induction of alternative isoforms by E + P. **a** The alternative splicing events including skipped exon, 5′ and 3′ alternative splice site, mutually exclusive exons, and retained intron were discovered in the RNA-seq data across the three cell lineages, using rMats package. MaSC, LP, and LM cell lineages derived from FVB mammary gland after exposure to EP. Pathway enrichment was performed to examine enriched pathways in the cell lineages using the online bioinformatics tool DAVID in differentially spliced genes (FDR < 0.1). Total enrichment score of the GO pathways as contributed by the different alternative splicing events in plotted for **b** MaSC, **c** LP, and **d** LM in the comparison between EP-treated mice and sham group

A query into splicing events using rMats [44–46] revealed five types of splicing events: skipped exon, alternative 5′ splice site, alternative 3′ splice site, mutually exclusive exon, and retained intron. The frequency of each of these events in the MSC, LP, and LM treated with EP and compared to control group is provided in Fig. 4a. Supplementary Tables 5, 6 and 7 list unique splicing events for MSC, LP, and LM subpopulations. In order to identify PR-mediated alternative splicing events, we examined the splicing events in the EP + TPA, and EP + MFP treatment groups. The events common between EP + TPA and EP + MFP treatment groups in MSC (Supplementary Table 8), LP (Supplementary Table 9), and LM (Supplementary Table 10) cells were filtered for FDR < 0.1 and only the splicing events repressed by the PR inhibitors were included in the enrichment examination. Pathway enrichment analysis of the genes differentially spliced (FDR < 0.1) between EP versus sham-treated groups and mediated by PR (i.e., spliced in EP-treated mice but not in EP + PR inhibitor-treated mice) were analyzed using DAVID [39], which revealed enrichment of similar pathways across the MSC, LP, and LM lineages. The enrichment score for each GO term was plotted with the contribution of each type of splicing event for the three lineages. All three lineages showed enrichment in cell-cell adhesion, cell cycle, DNA binding, and RNA splicing (Fig. 4b–d).

### Regulatory motifs associated with RNA binding proteins and splicing factors in response to progesterone exposure

**Fig. 5:**
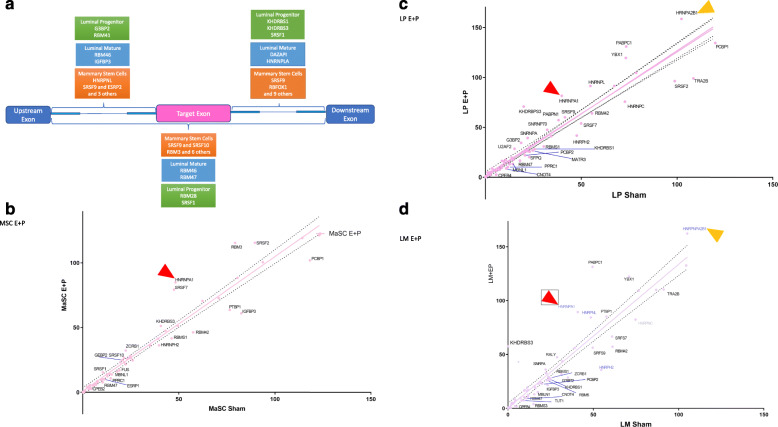
Regulatory motifs associated with RNA-binding proteins and RNA-binding proteins/splicing factors expressed in response to E + P. **a** RNA-binding protein motifs involved in alternative splicing were discovered using rMAPs. Differentially spliced genes (FDR < 0.1) in MaSC, LM, and LP lineages treated with EP relative to sham were examined for the motifs enriched in the alternatively spliced regions. RPKM values of the RNA-binding proteins were examined for correlation between EP-treated mice and the mice in control group in all the three cell lineages, **b** MaSC, **c** LP, and **d** LM. HNRNPA1 is indicated by the red triangle, HRNPA2B1 by the gold triangle

The motifs associated with RNA-binding protein (RBP) adjacent to splice sites found to be enriched in the EP group (Supplementary Tables 11, 12 and 13) were matched with the motifs downregulated in groups treated with EP + TPA and EP + MFP (Supplementary Tables 14, 15 and 16) to establish a specific PR-mediated response. Figure 5a gives a detailed account of the RBP associated with motifs found enriched in MSC (Supplementary Table 11), LP (Supplementary Table 12), and LM (Supplementary Table 13) cells in response to EP (FDR < 0.1). Our data revealed an enrichment of genes encoding proteins in the RBM and SRSF families. Figure 5b shows the correlation between expression values for the RNA-binding proteins in the MSC compartment in EP-treated and control groups. A higher expression of *SrsF* and *Rbm* family members *Srsf2*, *Srsf7*, and *Rbm3* was observed in the EP-treated group and, conversely, downregulation in the cells derived from mice treated with EP + PR inhibitors (data not shown), indicating a specific PR-mediated response. Various members of the *Snrnp*, *Srsf*, *Rmb*, and *Hnrnp* families showed induction of expression mediated by EP in LP cells (Fig. 5c). In the LM cells, an increase in the expression of *Hnrnp* family genes (Fig. 5d) was observed upon EP exposure. Common to all three subpopulations was the increase in *Hnrnpa1* expression and common to the luminal compartments was the increased expression of *Hnrnpa2b1* following EP exposure. These results indicate that the expression of a number of genes encoding RNA binding proteins are modulated by EP.

### Progesterone receptor regulates the alternative splicing

Since there were splicing events unique to mice treated with EP (Supplementary Tables 5, 6 and 7) and different sets of splicing events (Supplementary Tables 8, 9 and 10) discovered in mice treated with EP + PR inhibitors, it can be hypothesized that EP, in a PR-mediated manner, regulates splicing of a number of genes. We attempted to validate our alternative splicing results using an orthogonal methodology, i.e., digital drop PCR. Unfortunately, there was insufficient RNA remaining after sequencing for comparisons of treated cell subtypes, i.e., LPs and MSCs. Therefore, we retrieved published data in which cell lineages could be determined and in which groups differed by progesterone exposure. We chose to utilize the data developed by Bach et al. [48], who performed single cell sequencing of mouse mammary epithelial cells at four time points: Nulliparious, 14.5 days gestation, 6 days lactation, and 11 days post natural involution. Progestone levels are at a maximum at 14.5 days gestation in mice and, depending on the specific mouse strain, average 3× to 5× the concentration at day 1; both estradiol and testosterone concentrations are maximal days later [56]. Comparing nulliparous to 14.5 day gestation and using this as a surrogate for CvEP, we were able to validate specific splices in thirteen genes in the luminal compartment: *Cdh1*, *Srrm2*, *Enah*, *Prpf40a*, *Pik3c2a*, *Brd4*, *Card19*, *Emc10*, *Golga1*, *Nop58*, *Trp53inp1*, *Wipi1*, and *1110032A03Rik* and in one in the MSC compartment: *Eif4a* (Supplementary Table 17)*.*

### Alternative splicing effects genes involved in EMT

**Fig. 6:**
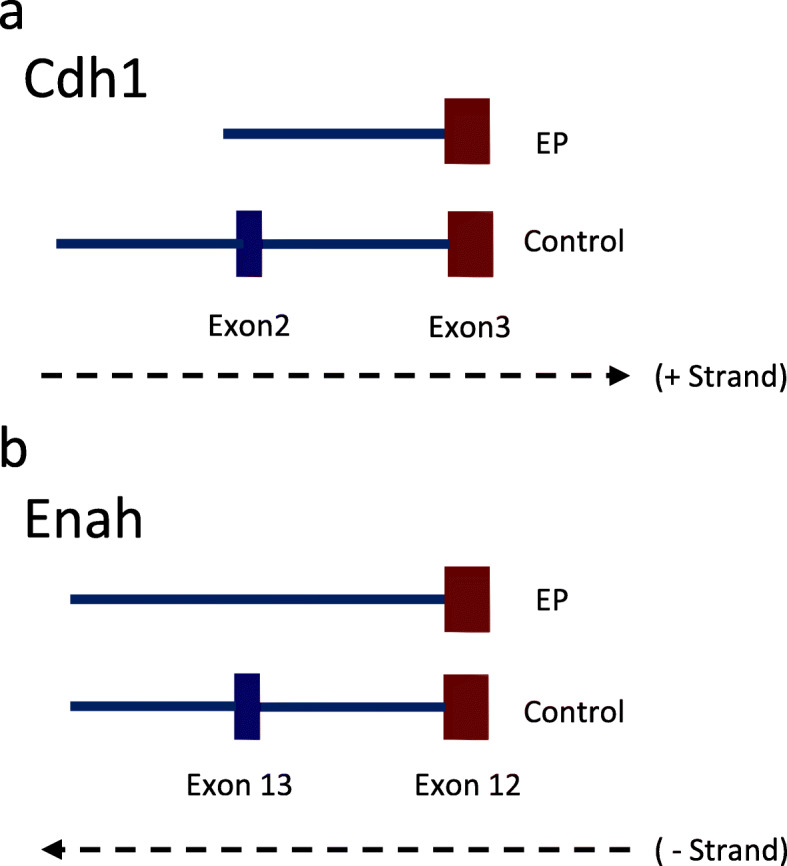
Alternative splicing of EMT-associated genes. EMT-associated genes were examined for gene-specific events after being filtered for FDR (0.1). Skipped exon 2 in **a** *Cdh1* discovered in response to EP, this event was observed in LP cells upon exposure to EP but not in LP treated with EP and PR inhibitors. **b** *Enah* displayed skipped exon 13/16 in EP-treated LP cells, this effect was not reversed in the group treated with EP and PR inhibitors. Note: This exon, by amino acid sequence, is identical to human exon 11a

Having observed EMT-like gene expression upon EP exposure and the enrichment of cell-cell adhesion pathways in the skipped exon events, we decided to examine the candidate EMT-associated genes for alternative splicing. Individual EMT-associated genes were queried for splicing events in the alternative splicing data filtered by FDR < 0.1. Figure 6a gives a UCSC Genome Browser view of the skipped exon in the *Cdh1* gene in the EP-treated group. Exon 2 was skipped in *Cdh1* in LP cells treated with EP but not in the counterpart treated with EP + MFP or EP + TPA indicating mediation by PR. Figure 6b provides the UCSC Genome Browser view of the skipped exon in *Enah* in the EP-treated group. Exon 13 is present in the LP controls but skipped in the EP-treated group.

## Discussion

Recent studies have linked progesterone exposure to events that promote neoplastic transformation both in humans and mice. One such phenomenon is expansion of the stem cell pool. With the eventual goal of applying PR inhibitors in the breast cancer prevention arena, we have examined the effects of PR blockade on mammary cell lineages in the mouse mammary gland, following exposure to estrogen plus progesterone. We confirmed the previous reports of EP-induced MSC expansion [3, 57] and demonstrated that this expansion was reversible with the use of PR inhibition. The reversal of MSC expansion was observed equally with both TPA and MFP and was accompanied by inhibition of paracrine signaling by *Wnt4* and *RANKL* in the LP cells. We observed a significant induction of a cell adhesion/EMT-like profile with EP exposure, and a downregulation of EMT-related transcription factors (*Zeb1*, *Zeb2*, *Snai1*, and *Gli3*) with PR inhibitor treatment, particularly in the MSC and LP compartment.

Our results are significant in the context that stem cells are a self-renewing population, which undergo expansion upon progesterone exposure in luteal phase in women and with each pregnancy. Tomasetti and colleagues contend that much of cancer risk can be attributed to the number of stem cell divisions within a tissue [9, 10]. Among all tissues, only skin, by their calculations, has a greater number of lifetime cell divisions than the breast. It is estimated that approximately three mutations occur every time a normal human stem cell divides [58, 59]; each an opportunity for a effector gene mutation. Our results clearly demonstrate that EP-driven MSC expansion is suppressed by PR blockade. Asselin-Labat et al. demonstrated a similar effect by attenuating ER signaling using an aromatase inhibitor [2], but did not observe this with MFP, likely related to use of a lower dose than was used in our experiments. In order to test the hypothesis of a dose effect, our mouse experiment will need to be repeated and both doses of MFP included. Tomasetti and Vogelstein [10] argue that a strategy of restraining stem cell division for cancer prevention is limited by potential deleterious consequences on tissue development and maintenance. That, however, may not be the case for the breast where, once breast development in puberty is complete, stem divisions could be intermittently placed on hold by antiprogestins.

The effects of progesterone are context dependent. Progesterone can elicit either proliferative or antiproliferative effects on breast epithelial cells that depends, for example, on the experimental model system [60], normal breast versus progressive breast cancer, the duration of treatment, and dose [61]. Treatment with high doses of non-metabolizable synthetic progestins produces sustained autoinhibition, due in part to the fact that liganded PR induces expression of the cell cycle inhibitors, p21 and p27 [61, 62]. Prior to the introduction of the myriad effective antiestrogen therapies, high-dose progestins were utilized in the second line as therapy for hormone-responsive breast cancers [63]. In the normal, healthy breast, it is posited that progesterone works by binding to PR in the minority of PR+ cells [64]. Translocation to the nucleus and recruitment of cofactors enables the transcription of paracine factors that stimulate proliferation of hormone receptor-negative cells in the basal/MSC as well as luminal progenitor compartments [8]. During oncogenic progression, the PR+ cells are thought to switch from paracrine to an autocrine regulation of proliferation and even later in the progression, progesterone inhibits various aspects of tumorigenicity including invasion and metastasis, hence its use earlier as a second-line therapy [64]. While we posit that the expansion of the MSC compartment is due to paracrine effectors eliciting proliferation in hormone receptor-negative cells, we note that Hilton and colleagues identified a bipotent progenitor residing in the basal compartment that expresses PR but not ER [65]. This was also observed by Arendt et al. in a separate study [66]. These cells would be theorized to be responsive to P4 and, therefore, could be the cells that are responsible for the increase in the MSC that we observe. However, interrogation of snATAC-seq data in search of basal cells in the adult mammary gland correlating to bipotential progenitors, which have not been observed in lineage-tracing studies, did not reveal such a population [67]. Therefore, the possibility that our MSC results are due to the proliferation of such cells must remain a hypothesis to be tested.

Our selection of MSC, luminal progenitor (CD24^+^CD49f^lo^CD61^+^) and luminal mature populations (CD24^+^CD49f^lo^CD61^−^) was consistent with previous reports [2, 3, 8]. Using the same gating strategy, Joshi and colleagues observed the same increase in the CD24^+^CD49f^lo^CD61^−^ (LM) fraction and decrease in CD24^+^CD49f^lo^CD61^+^ (LP) as we did. However, a different combination of markers enabled them to distinguish ER^+^PR^+^ nonclonogenic, ER^+^PR^+^ luminal progenitor, and ER^−^PR^−^ luminal progenitor lineages (gating #3 )[8], revealing an increase in the ER^−^PR^−^ luminal progenitor compartment with EP exposure. Further examination has established that the progesterone-driven loss of luminal progenitors is limited to the CD61^+^ subset [68] which is consistent with our results.

In agreement with prior reports pointing to paracrine signaling from the luminal compartment as the instigator of proliferation in the MSC population [2, 8], we observed significant induction of *Wnt4* and *Rankl* in the LP cells, which was blocked by both MFP and TPA. The progesterone-induced *Wnt* response requires RANKL-RANK signaling and is enhanced by RANKL-RANK induced *Rspo1* expression [8], which we find is increased by EP exposure in the LM compartment (Fig. 2). Additionally, *Axin2*, a Wnt target gene is increased by EP in the basal and luminal progenitor compartments [8]. We observed the greatest increase of *Axin2* expression in response to EP in the LP fraction with a lesser but statistically significant increase in MaSc; this is blocked in both compartments by the antiprogestins. Additionally, we saw abrogation by TPA and MFP of the increased expression of the *Wnt* receptor *Lrp5* in all three compartments. Our results clearly demonstrate that the paracrine effects of progesterone can be blocked in the LP by antiprogestins.

*MMP7* activation is downstream of the *WNT4*/*CTTNB1* pathway in the human mammary gland [69, 70]. It is well known that *Mmp7* can target E-cadherin for degradation [71], the loss of which is central to EMT. Examination of our RNA seq data for enrichment of EMT-associated genes revealed downregulation of *Cdh1* and induction of *Zeb1*, *Zeb2*, *Snai1*, and *Gli3* transcription factors. Transcription factors *Zeb1*, *Zeb2*, and *Snai1* are recruited to *Cdh1* promoter to repress its expression. A previous report showing induction of *Zeb1* in the normal human mammary gland in luteal phase is consistent with our observation [26]. The antiprogestins that we tested blocked the majority of changes in EMT-related gene expression that we observed in the EP treatment group.

Previous reports have demonstrated that splicing events in EMT genes can play an important role in acquisition of mesenchymal-like profile [72]. Our data revealed exon skipping in *Cdh1* and *Enah*, which may influence EMT. *ENAH11a* (Mena11a) is an isoform of *ENAH* that is epithelial specific and located at tight junctions and adherens junctions [73]. It is excluded during EMT [74].

Exon 2 of Brd4 is skipped in the ER-treated LP cells. An examination of splice isoforms in Ensmbl (ENSMUSG00000024002) reveals that two of the isoforms missing Exon 2, Brd4-205, and Brd4-212 have an incomplete 3′ coding sequence. This is an important observation as the short form of the human BRD4, which lacks 640 amino acids at the 3′ end is oncogenic in breast cancer, whereas the long form acts as a tumor suppressor [75]. The short isoform has been identified to bind with En1 to enhancers of specific matrisome genes [75] or act as an endogenous inhibitor of the DNA damage response [76].

RNA-binding protein motifs discovered adjacent to splice sites in the MSC cells derived from mice treated with EP showed an overlap with genes encoding EMT-associated RNA-binding proteins *Esrp2* and *Rbfox1* [21]. The same motifs were found repressed relative to control in the MSC derived from mice treated with EP + PR inhibitors. HNRNPAB is a progesterone-responsive gene [77]. Hnrnpa2b1 expression was observed to be induced by EP in both luminal subpopulations. It regulates the alternative splicing of the RON receptor tyrosine kinase in the U87MG glioblastoma cell line and increases its translation in breast cancer cells [78, 79]. This splicing factor has a number of targets in glioma, at least some of which are likely to be involved in EMT as its suppression has been shown to inhibit both EMT and invasion [78, 79]. In our mouse model, *Hnrnpa1* expression is increased with EP exposure in all three lineages of the mammary gland [21]. *Hnrnpa1* controls the splicing of a wide variety of transcripts and is highly expressed in breast cancers, where it is associated with metastatic progression [80, 81]. Another RNA-binding protein, RMB3, shows increased gene expression in the MSC compartment in response to EP. In colon cancer cells, this protein increases stem cell marker and increases the expression of the Wnt/β-catenin pathway member *LRG5*. This potentially is another means by which PR-mediated signaling is affecting stem cell numbers via Wnt/β-catenin signaling [82].

## Conclusions

Epidemiological and laboratory studies have revealed a complex role for progesterone in the mammary gland, with evidence mounting that this likely plays a role in creating a breast cancer permissive environment. This pro-tumorigenic effect has been partially attributed to the effect of progesterone on mammary stem cell (MSC) expansion, prompting us to inquire into the ability of progesterone receptor (PR) inhibitors to reverse this effect. In sum, our experiments have demonstrated that the blockade of progesterone’s binding to its receptor by the antiprogestins TPA and MFP in the murine mammary gland eliminates MSC and LM cell expansion most likely via the interruption of paracrine signaling. Additionally, we provide evidence of an expanded portfolio of EP activities that are also affected by PR blockade including EMT, the expression of RNA-binding proteins and alternative RNA splicing. These finding have profound implications for breast cancer prevention and treatment.

## Supplementary Information

**Additional file 1 **Supplementary Figure 1. Workflow of isolation of the mammary epithelial and stromal cells. An Inguinal and thoracic gland were isolated from 10-week-old FVB mice after 14 days of treatment with either Sham; EP; EP+ TPA; EP + MFP. Isolated cells were digested into a single cell population, which was labelled with respective markers for sorting into sub populations of mammary stem cells (MaSC, CD61^+^CD49f^hi^), luminal progenitor (LP, CD61^+^CD49f^lo^) cells and luminal mature cells (LM, CD61^-^CD49f^lo^) cells. RNA was isolated from sorted cells and sent for library preparation and RNA sequencing. Sequenced files were aligned to the mouse genome and examined for differential gene expression and alternative splice variant analysis. Differential gene expression was validated by qPCR for candidate genes and enrichment analysis. Alternative splice variant analysis included examining splicing events in candidate genes, and determining the RNA-binding proteins (RBP) and splicing factors expressed upon exposure to EP. The identity of cell lineages isolated was confirmed using qRTPCR for transcriptional markers. Livak’s fold change was calculated relative to MaSC (sham) cell population. (b) *Krt14* and (c) *Sma* had higher expression in MaSC cell population compared to the cells in the luminal compartment. High expression of markers for luminal cells, (d) *Gata3* and (e) *Krt18* was observed in LP and LM cells.

**Additional file 2.** Supplementary Figure 2. Sorting plan for FACS. After removing hematopoietic and endothelial cells, CD24 and CD49 were employed to define the basal epithelial population (BPOP; CD24^+^CD49f^hi^) and the luminal population (LPOP; CD24^+^CD49f^low^). Using CD61, cell lineages were further defined into MSCs (CD61^+^CD24^+^CD49f^hi^), LP cells (CD61^+^ CD24^+^CD49f^lo^), and LM cells (CD61^-^ CD24^+^CD49f^lo^). Percentages were calculated as: MSCs/BPOP; LP/LPOP and LM/LPOP.

**Additional file 3.** Supplementary Figure 3. scRNA and Bulk RNA-seq Alternative Splicing Analysis Workflow. Lefthand flow: Significant alternative splicing events occurring exclusively in the LP or MSC cells were identified using rMATs. Righthand flow: FASTQ files from Bach et al. [48] were downloaded and Cell Ranger utilized to generate .bam and .cloupe files. Subsequently, cell transcriptomes were clustered independently for each replicate and developmental stage to delineate major cell groups using k-means clustering. For each resulting cluster, the mean expression of Krt18 and Krt5 and the proportion of positive cells was tabulated. Based on these data, cells were designated as belonging to Krt5-high, Krt18-high, Krt18-low and ‘other’ clusters. Independent .bam files for each cluster type based on the cell name/barcode were generated. The resulting cell type, LC or BC, and stage-specific, Nulliparous (NP) or Gestational (G), .bam files were then re-mapped to GRCm38 to generate SJ.out.tab files containing splice junction reads for analysis with Outrigger. The significant and unique AS events from rMATs were then compared to the Krt18-high (luminal) and Krt5-high (basal) Outrigger results from each stage based on genomic coordinates (with a buffer +/- 20 base pairs). Note: Outrigger identifies only skipped exons or mutually exclusive exon events.

**Additional file 4 **Supplementary Figure 4. scRNA-sequencing Clustering. Clustering of scRNA-sequencing data was implemented in order to identify luminal and basal cell lineages *post hoc*. FASTQ files from Bach et al. [48] were used to generate .bam and .cloupe files using *CellRanger* [see methods]. Both tSNE and UMAP dimension reductions were used for 2-dimensional visualization. *Loupe Browser* was used to visualize each replicate and developmental stage, defined by Bach et al., to delineate major cell groups using K-means clustering (Supplementary Fig. 4 a, d, g, j, m, p, s, v). From each resulting cluster, we calculated the mean expression of *Krt18* & *Krt5* and the proportion of positive cells. Based on these data points, cells were designated to one of four clusters: *Krt5*-high, *Krt18*-high, *Krt18*-low, and ‘other’ for non-specific expression values (*Krt18* clusters: Supplemantary Fig. 4 c, f, i, l, o, r, u, x; *Krt5* clusters: Supplementary Fig. 4 b, e, h, k, n, q, t, w). The *Subset-bam* tools (methods) was then used to generate independent .bam files for each cluster type based on cell name/barcode extracted from *Loupe Browser* clustering. Note: Cluster ID’s, i.e: cluster 1, cluster 2, etc., found in K-means (Supplementary Fig. 5 a, d, g, j, m, p, s, v) should be used to identify specific *Krt18*-high, *Krt18*-low, and *Krt5*-high clusters.

**Additional file 5.** Supplementary Figure 5. Pathway enrichment analysis. Pathway enrichment was examined using the online tool DAVID, and the enrichment of GO pathways was plotted for the three cell lineages. Enriched GO pathways were examined for the differentially expressed genes between Sham and EP & EP + PR inhibitors (TPA and MFP) in MaSC (Sup. Fig. 5a), LP (Sup. Fig. 5b), LM (Sup. Fig. 5c). The enrichment of GO terms was examined using DAVID; the enrichment score of over represented pathways from the differentially expressed gene list is relative to the genes that were not differentially expressed. MaSc cells (Sup. Fig. 1a) showed the enrichment of PI3K pathway, cell cycle proteins, cell adhesion molecules and WNT signaling in response to EP and a repression after in the group treated with EP + PR inhibitors. These results are concordant with the proliferative response to EP observed in MaSC cells. In the LP cells (Sup. Fig. 1b) each of the pathways were enriched by E + P but the WNT pathway stimulation was approximately twice as great as the other pathways and only the WNT pathway was suppressed by PR inhibitor treatment. In LM cells (Sup. Fig. 1c) PI3K-Akt signaling cell cycle protein, WNT signaling and cell adhesion molecules were enriched similarly to the same pathways in MaSC cells in response to EP and were correspondingly repressed by treatment with EP + PR inhibitors.

**Additional file 6.** Supplementary Figure 6. EMT gene expression in the mouse mammary gland. (a) Gene expression of selected EMT genes upon exposure to EP or EP + TPA. qRTPCR was conducted to examine estrogen receptor and progesterone receptor expression in response to EP or EP + PR inhibitors. (b) PR in MaSC, LP and LM cells after treatment in sham, EP, EP + TPA and EP + MFP and (c) ESR1 in LM after treatment with sham, EP, EP + TPA and EP + MFP.

**Additional file 7.** Supplementary Table 1: list of primers utilized

**Additional file 8.** Supplementary Table 2: List of differentially expressed genes, MSC

**Additional file 9.** Supplementary Table 3: List of differentially expressed genes, LP

**Additional file 10.** Supplementary Table 4: List of differentially expressed genes, LM

**Additional file 11.** Supplementary Table 5: Alternative splicing, EP, MSC

**Additional file 12.** Supplementary Table 6: Alternative splicing, EP, LP

**Additional file 13.** Supplementary Table 7: Alternative splicing, EP, LM

**Additional file 14.** Supplementary Table 8: Alternative splicing, EP + PR inhibitors, MSC

**Additional file 15.** Supplementary Table 9: Alternative splicing, EP + PR inhibitors, LP

**Additional file 16.** Supplementary Table 11: Alternative splicing, EP + PR inhibitors, LM

**Additional file 17.** Supplementary Table 11: RNA binding protein motifs, EP, MSC

**Additional file 18.** Supplementary Table 12: RNA binding protein motifs, EP, LP

**Additional file 19.** Supplementary Table 13: RNA binding protein motifs, EP, LM

**Additional file 20.** Supplementary Table 14: RNA binding protein motifs, EP + TPA, MSC

**Additional file 21.** Supplementary Table 15: RNA binding protein motifs, EP + TPA, MSC

**Additional file 22.** Supplementary Table 16: RNA binding protein motifs, EP + TPA, MSC

**Additional file 23.** Supplementary Table 17: Validated alternative splicing

## Abbreviations

ANOVA: Analysis of variance
AS: Alternative splicing
BCa: Breast cancer
Chip: Chromatin immunoprecipitation
E: Estradiol
EMT: Epithelial to mesenchymal transition
ER: Estrogen receptor
FDR: False discovery rate
FACS: Flow-assisted cell sorting
FVB: Friend virus B
GO: Gene ontology
HR: Hormone receptor
LM: Luminal mature
LP: Luminal progenitor
MSC: Mammary stem cells
MFP: Mifeprestone
P: Progesterone
PR: Progesterone receptor
Q score: Quality score
RBP: RNA-binding protein
RIN: RNA integrity number
RNA-Seq: RNA sequencing
SPRM: Selective progesterone receptor modulator
TPA: Telapristone acetate
