# Supplementary figures and images for: Progesterone receptor antagonists reverse stem cell expansion and the paracrine effectors of progesterone action in the mouse mammary gland
Source: Breast Cancer Res. 2021 Aug 3;23:78. doi: 10.1186/s13058-021-01455-2 (PMC8330021; doi:10.1186/s13058-021-01455-2)

## Supplemental Figure 1

a

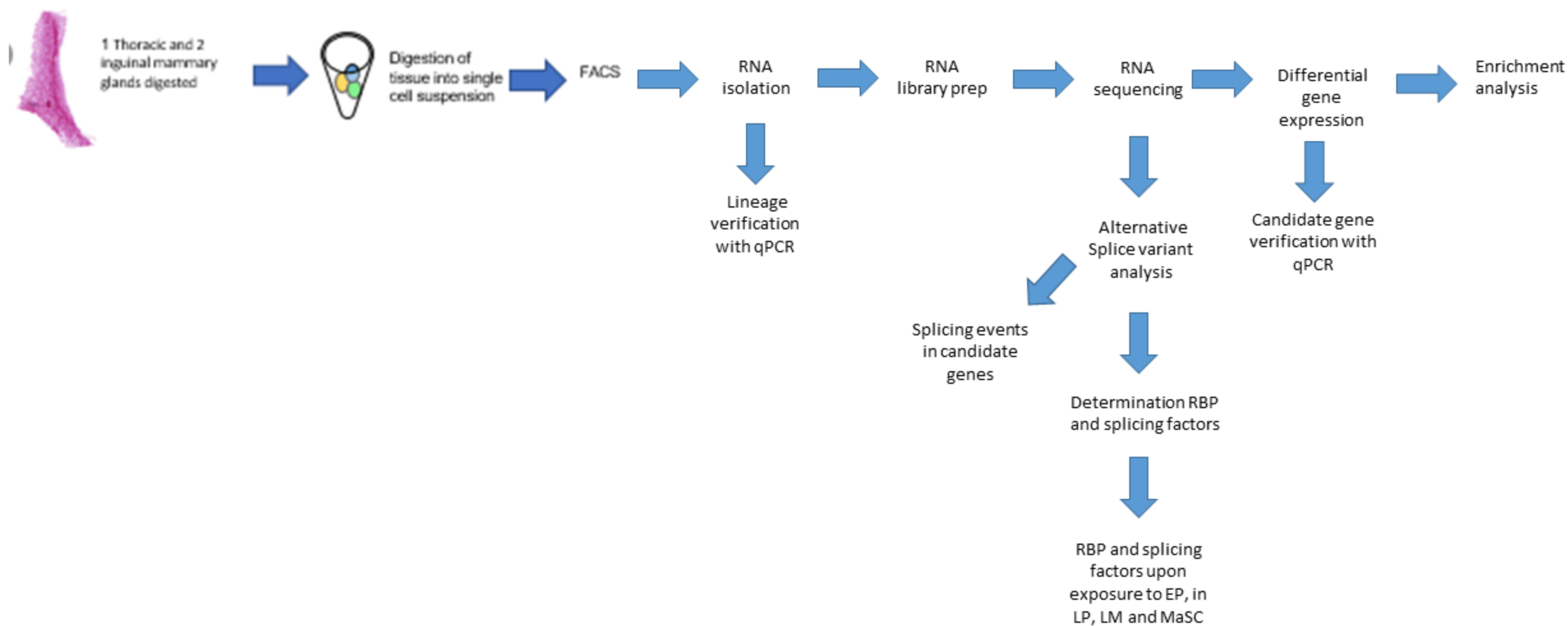

## Mammary Stem Cells

1b.

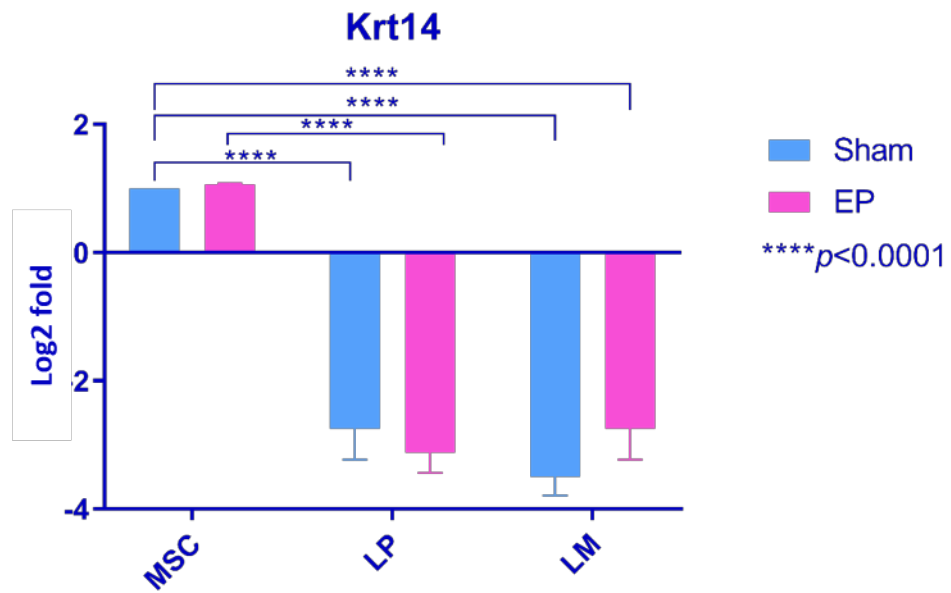

1c.

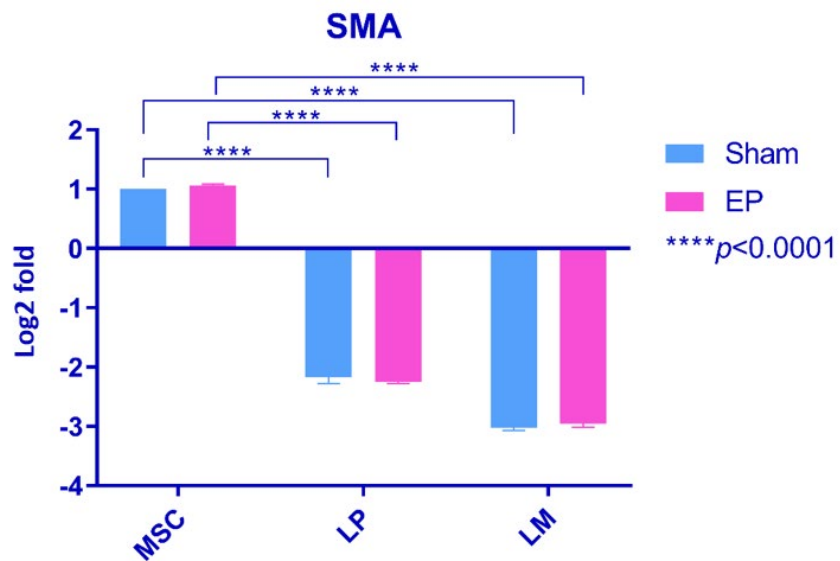

## Luminal Cells

1d.

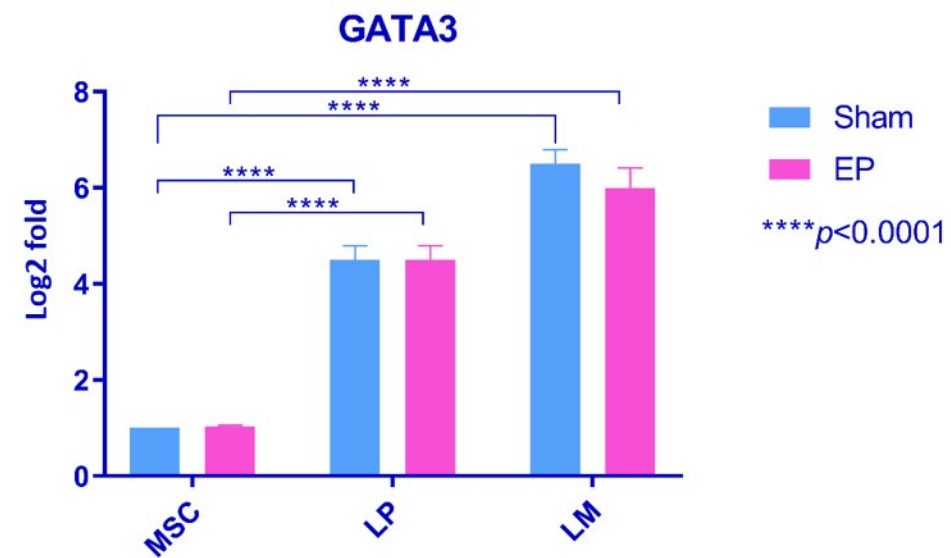

1e.

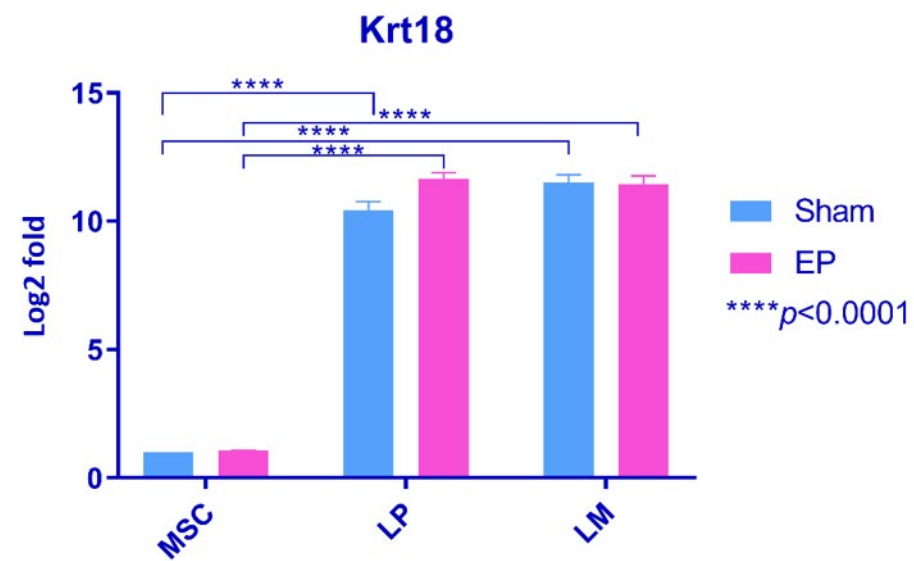

Supplement: Supplementary file 1 — Additional file 1 Supplementary Figure 1. Workflow of isolation of the mammary epithelial and stromal cells. An Inguinal and thoracic gland were isolated from 10-week-old FVB mice after 14 days of treatment with either Sham; EP; EP+ TPA; EP + MFP. Isolated cells were digested into a single cell population, which was labelled with respective markers for sorting into sub populations of mammary stem cells (MaSC, CD61+CD49fhi), luminal progenitor (LP, CD61+CD49flo) cells and luminal mature cells (LM, CD61-CD49flo) cells. RNA was isolated from sorted cells and sent for library preparation and RNA sequencing. Sequenced files were aligned to the mouse genome and examined for differential gene expression and alternative splice variant analysis. Differential gene expression was validated by qPCR for candidate genes and enrichment analysis. Alternative splice variant analysis included examining splicing events in candidate genes, and determining the RNA-binding proteins (RBP) and splicing factors expressed upon exposure to EP. The identity of cell lineages isolated was confirmed using qRTPCR for transcriptional markers. Livak’s fold change was calculated relative to MaSC (sham) cell population. (b) Krt14 and (c) Sma had higher expression in MaSC cell population compared to the cells in the luminal compartment. High expression of markers for luminal cells, (d) Gata3 and (e) Krt18 was observed in LP and LM cells. [file 13058_2021_1455_MOESM1_ESM.pdf]

## Cell Sorting Plan

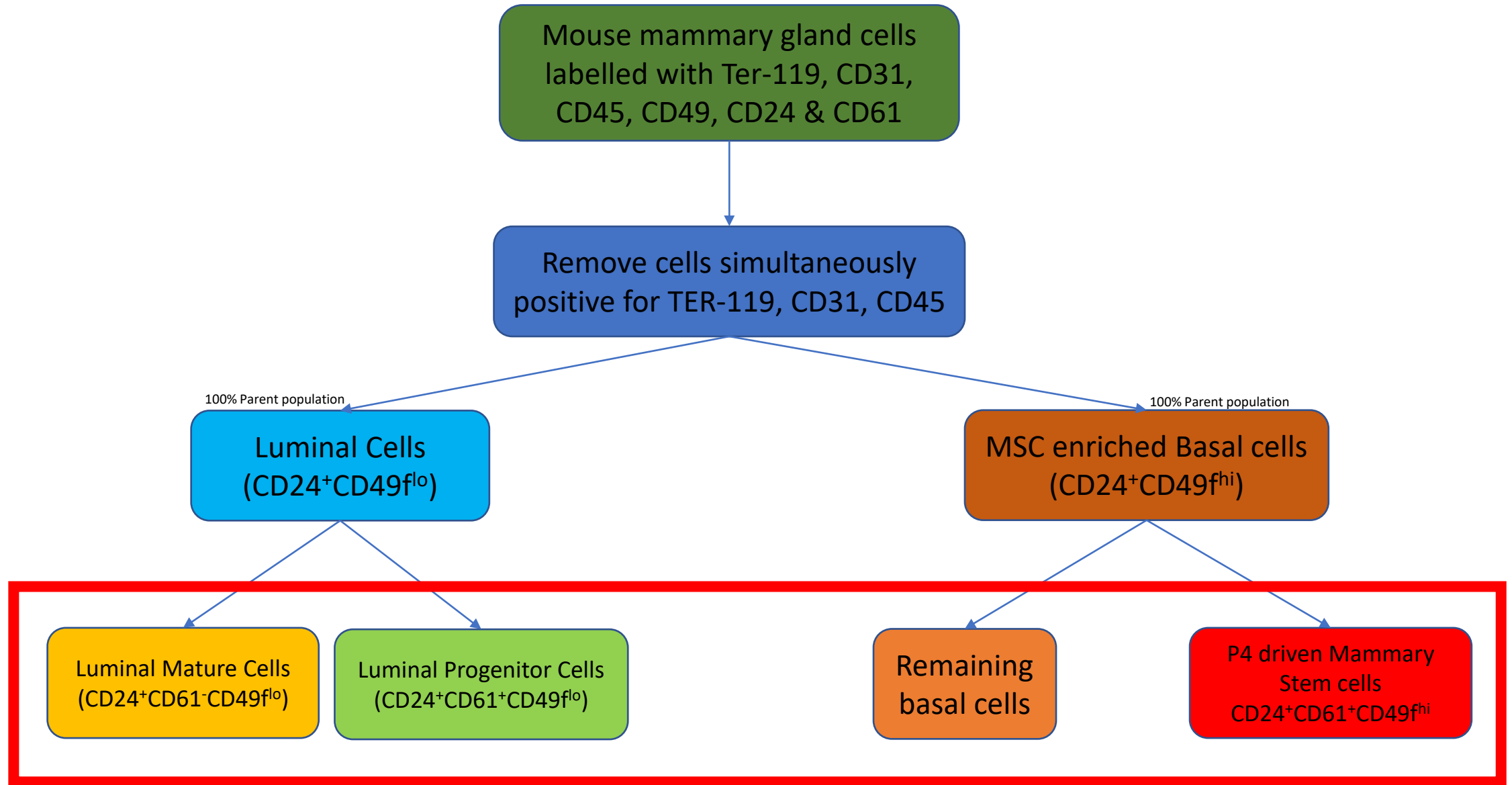

Supplement: Supplementary file 2 — Additional file 2. Supplementary Figure 2. Sorting plan for FACS. After removing hematopoietic and endothelial cells, CD24 and CD49 were employed to define the basal epithelial population (BPOP; CD24+CD49fhi) and the luminal population (LPOP; CD24+CD49flow). Using CD61, cell lineages were further defined into MSCs (CD61+CD24+CD49fhi), LP cells (CD61+ CD24+CD49flo), and LM cells (CD61- CD24+CD49flo). Percentages were calculated as: MSCs/BPOP; LP/LPOP and LM/LPOP. [file 13058_2021_1455_MOESM2_ESM.pdf]

# scRNA and Bulk RNA-seq Alternative Splicing Analysis Workflow

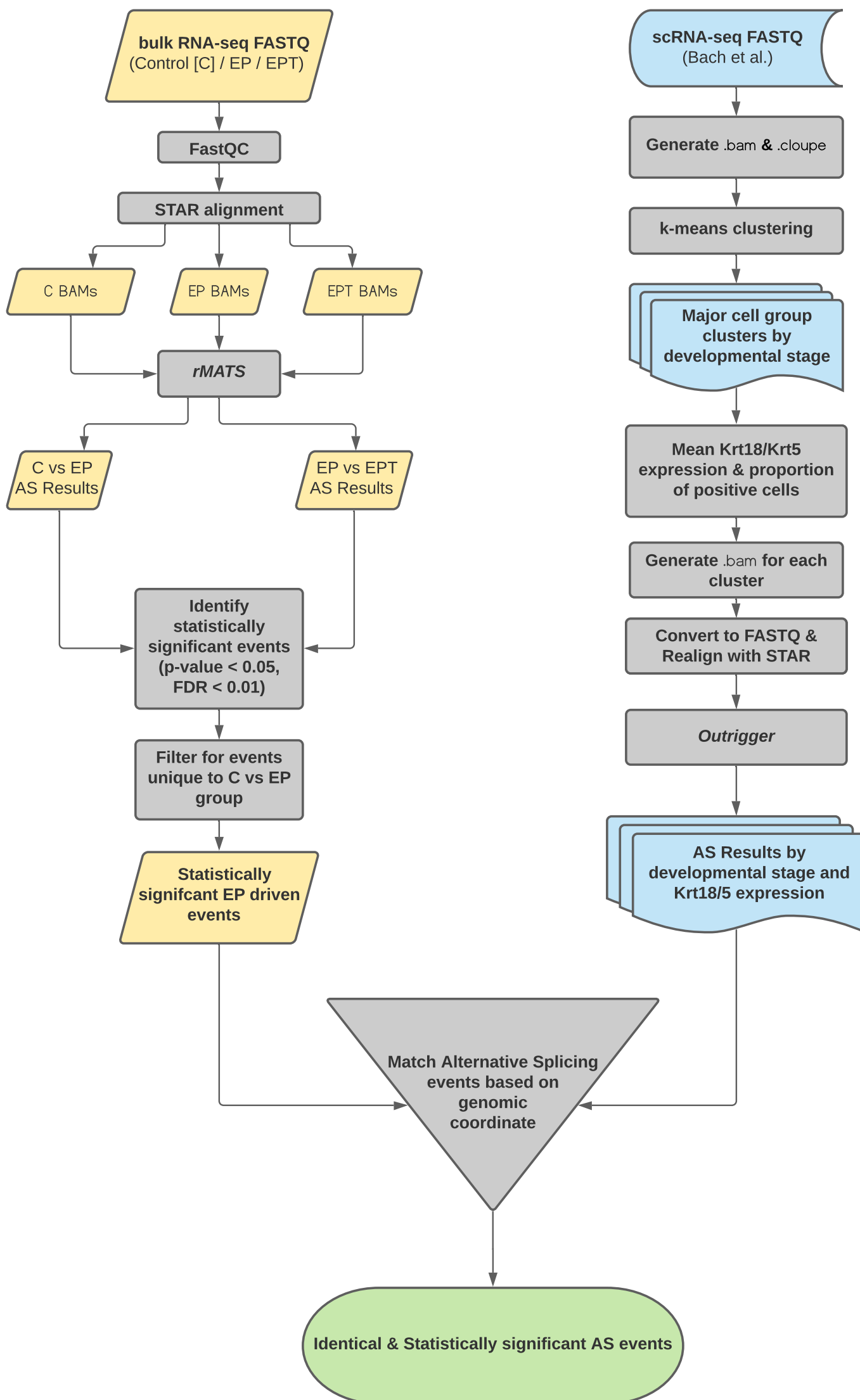

Supplement: Supplementary file 4 — Additional file 4 Supplementary Figure 4. scRNA-sequencing Clustering. Clustering of scRNA-sequencing data was implemented in order to identify luminal and basal cell lineages post hoc. FASTQ files from Bach et al. [48] were used to generate .bam and .cloupe files using CellRanger [see methods]. Both tSNE and UMAP dimension reductions were used for 2-dimensional visualization. Loupe Browser was used to visualize each replicate and developmental stage, defined by Bach et al., to delineate major cell groups using K-means clustering (Supplementary Fig. 4 a, d, g, j, m, p, s, v). From each resulting cluster, we calculated the mean expression of Krt18 & Krt5 and the proportion of positive cells. Based on these data points, cells were designated to one of four clusters: Krt5-high, Krt18-high, Krt18-low, and ‘other’ for non-specific expression values (Krt18 clusters: Supplemantary Fig. 4 c, f, i, l, o, r, u, x; Krt5 clusters: Supplementary Fig. 4 b, e, h, k, n, q, t, w). The Subset-bam tools (methods) was then used to generate independent .bam files for each cluster type based on cell name/barcode extracted from Loupe Browser clustering. Note: Cluster ID’s, i.e: cluster 1, cluster 2, etc., found in K-means (Supplementary Fig. 5 a, d, g, j, m, p, s, v) should be used to identify specific Krt18-high, Krt18-low, and Krt5-high clusters. [file 13058_2021_1455_MOESM4_ESM.pdf]

Supplemental Figure 5

a

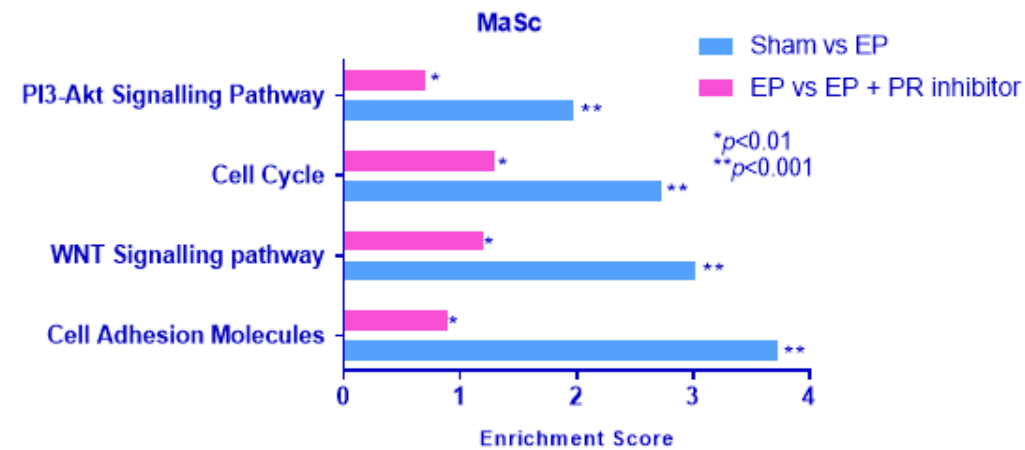

b

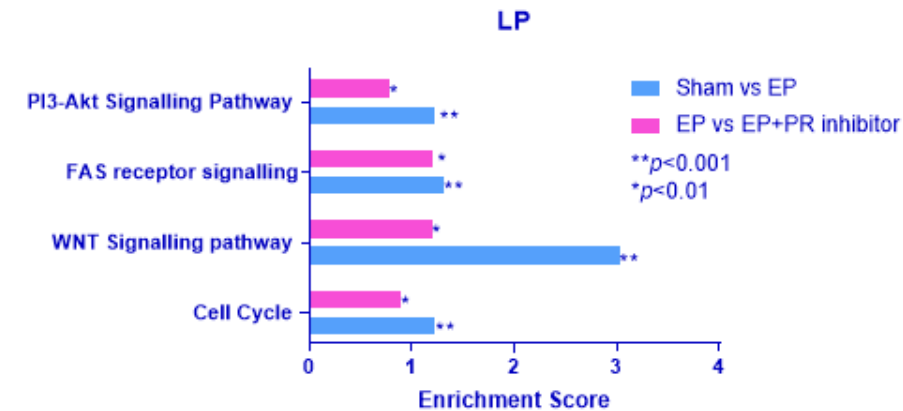

c

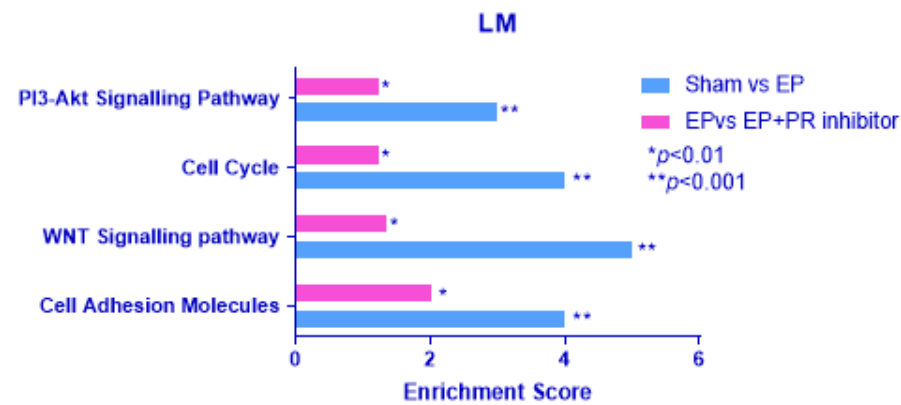

Supplement: Supplementary file 5 — Additional file 5. Supplementary Figure 5. Pathway enrichment analysis. Pathway enrichment was examined using the online tool DAVID, and the enrichment of GO pathways was plotted for the three cell lineages. Enriched GO pathways were examined for the differentially expressed genes between Sham and EP & EP + PR inhibitors (TPA and MFP) in MaSC (Sup. Fig. 5a), LP (Sup. Fig. 5b), LM (Sup. Fig. 5c). The enrichment of GO terms was examined using DAVID; the enrichment score of over represented pathways from the differentially expressed gene list is relative to the genes that were not differentially expressed. MaSc cells (Sup. Fig. 1a) showed the enrichment of PI3K pathway, cell cycle proteins, cell adhesion molecules and WNT signaling in response to EP and a repression after in the group treated with EP + PR inhibitors. These results are concordant with the proliferative response to EP observed in MaSC cells. In the LP cells (Sup. Fig. 1b) each of the pathways were enriched by E + P but the WNT pathway stimulation was approximately twice as great as the other pathways and only the WNT pathway was suppressed by PR inhibitor treatment. In LM cells (Sup. Fig. 1c) PI3K-Akt signaling cell cycle protein, WNT signaling and cell adhesion molecules were enriched similarly to the same pathways in MaSC cells in response to EP and were correspondingly repressed by treatment with EP + PR inhibitors. [file 13058_2021_1455_MOESM5_ESM.pdf]

Supplemental Figure 6

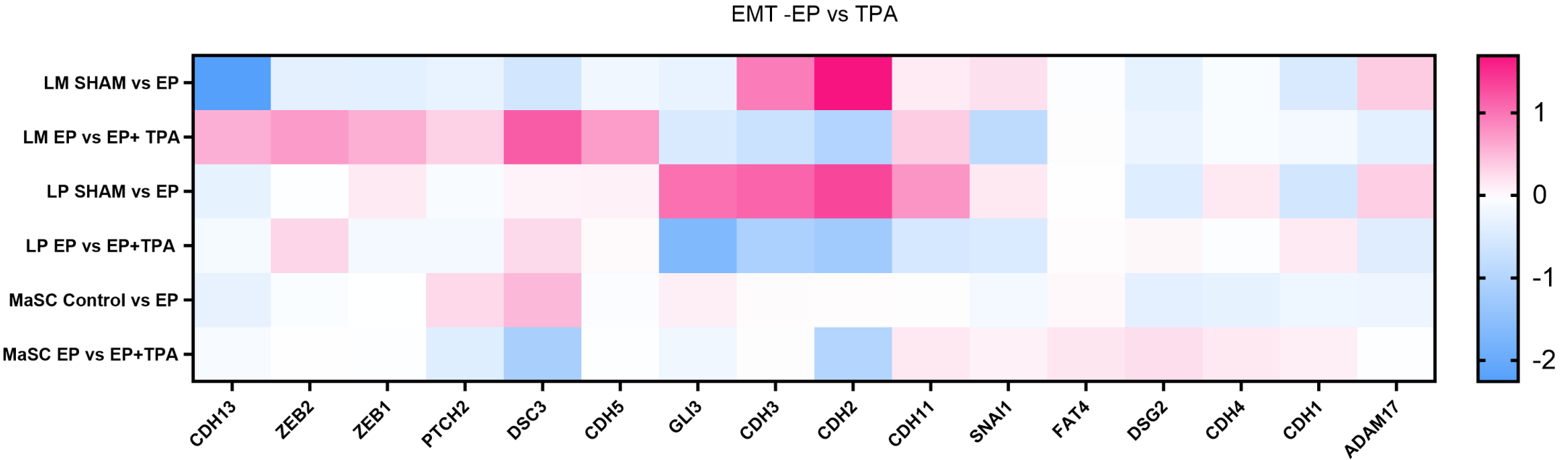

Supplement: Supplementary file 6 — Additional file 6. Supplementary Figure 6. EMT gene expression in the mouse mammary gland. (a) Gene expression of selected EMT genes upon exposure to EP or EP + TPA. qRTPCR was conducted to examine estrogen receptor and progesterone receptor expression in response to EP or EP + PR inhibitors. (b) PR in MaSC, LP and LM cells after treatment in sham, EP, EP + TPA and EP + MFP and (c) ESR1 in LM after treatment with sham, EP, EP + TPA and EP + MFP. [file 13058_2021_1455_MOESM6_ESM.pdf]
