# Supplementary material for: Progesterone receptor antagonists reverse stem cell expansion and the paracrine effectors of progesterone action in the mouse mammary gland
Source: Breast Cancer Res. 2021 Aug 3;23:78. doi: 10.1186/s13058-021-01455-2 (PMC8330021; doi:10.1186/s13058-021-01455-2)
Supplement: Supplementary file 3 — Additional file 3. Supplementary Figure 3. scRNA and Bulk RNA-seq Alternative Splicing Analysis Workflow. Lefthand flow: Significant alternative splicing events occurring exclusively in the LP or MSC cells were identified using rMATs. Righthand flow: FASTQ files from Bach et al. [48] were downloaded and Cell Ranger utilized to generate .bam and .cloupe files. Subsequently, cell transcriptomes were clustered independently for each replicate and developmental stage to delineate major cell groups using k-means clustering. For each resulting cluster, the mean expression of Krt18 and Krt5 and the proportion of positive cells was tabulated. Based on these data, cells were designated as belonging to Krt5-high, Krt18-high, Krt18-low and ‘other’ clusters. Independent .bam files for each cluster type based on the cell name/barcode were generated. The resulting cell type, LC or BC, and stage-specific, Nulliparous (NP) or Gestational (G), .bam files were then re-mapped to GRCm38 to generate SJ.out.tab files containing splice junction reads for analysis with Outrigger. The significant and unique AS events from rMATs were then compared to the Krt18-high (luminal) and Krt5-high (basal) Outrigger results from each stage based on genomic coordinates (with a buffer +/- 20 base pairs). Note: Outrigger identifies only skipped exons or mutually exclusive exon events. [file 13058_2021_1455_MOESM3_ESM.pdf]

a.

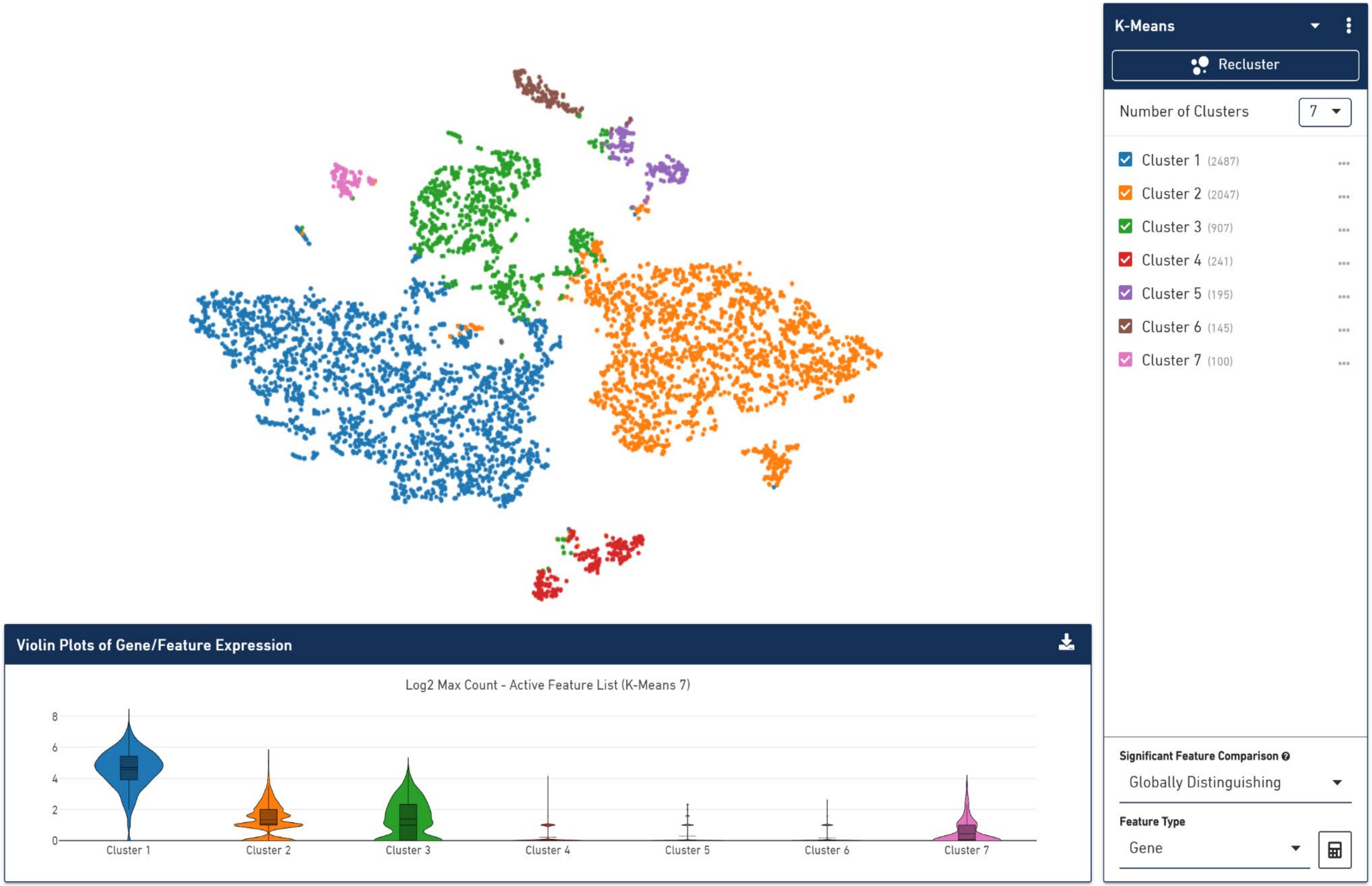

Stage: **Gestational**
Animal ID: **G1**
Clustering method: **tSNE**
Clustering: **K-means**

Cluster 1 = **Krt18 high**
Cluster 2 = **Krt5 high**
Cluster 3 = **Krt18 low**
Clusters 4-7 = **other**

b.

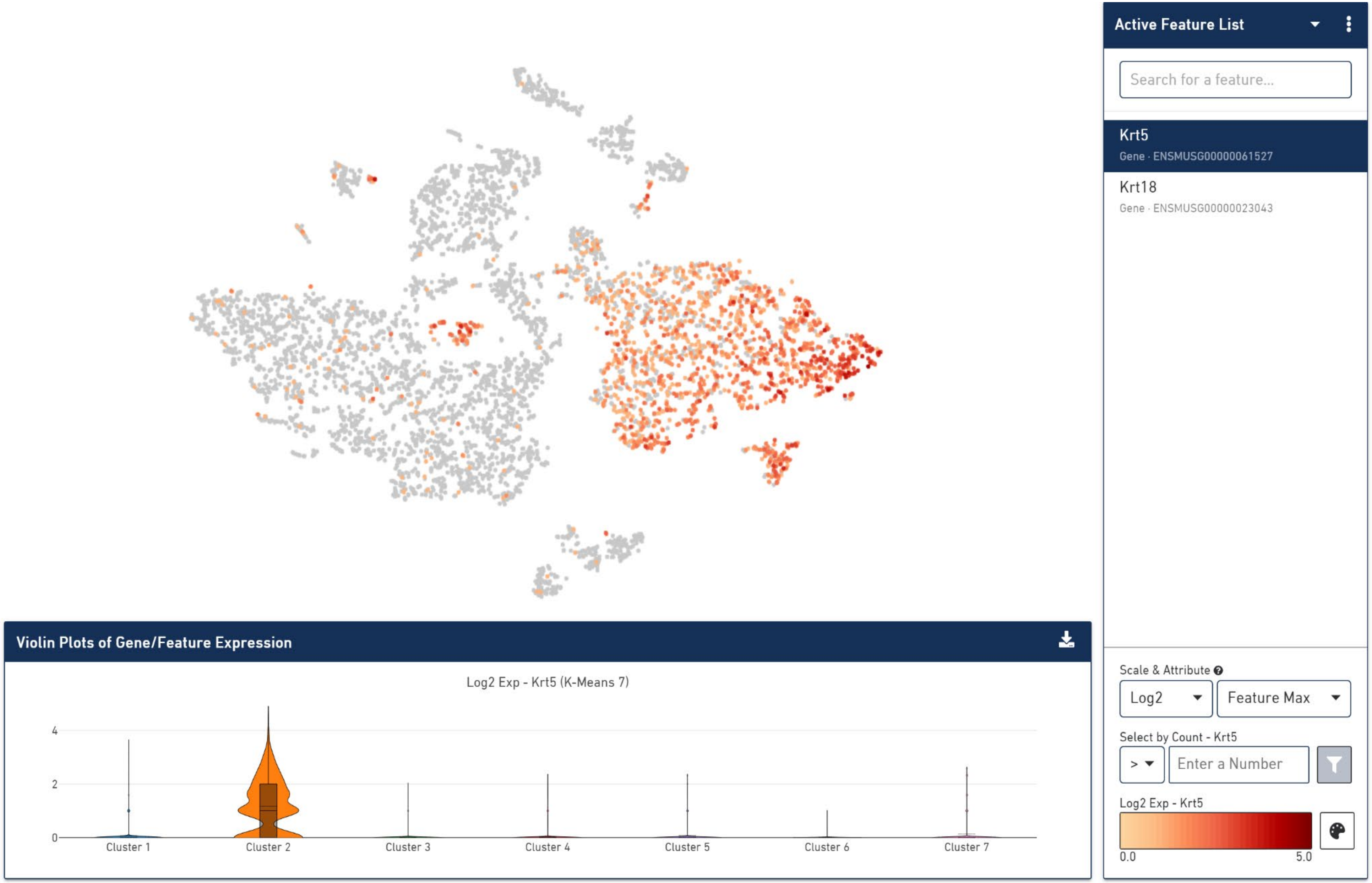

Stage: **Gestational**
Animal ID: **G1**
Clustering method: **tSNE**
Clustering: **Krt5**

Cluster 1 = **Krt18 high**
Cluster 2 = **Krt5 high**
Cluster 3 = **Krt18 low**
Clusters 4-7 = **other**

c.

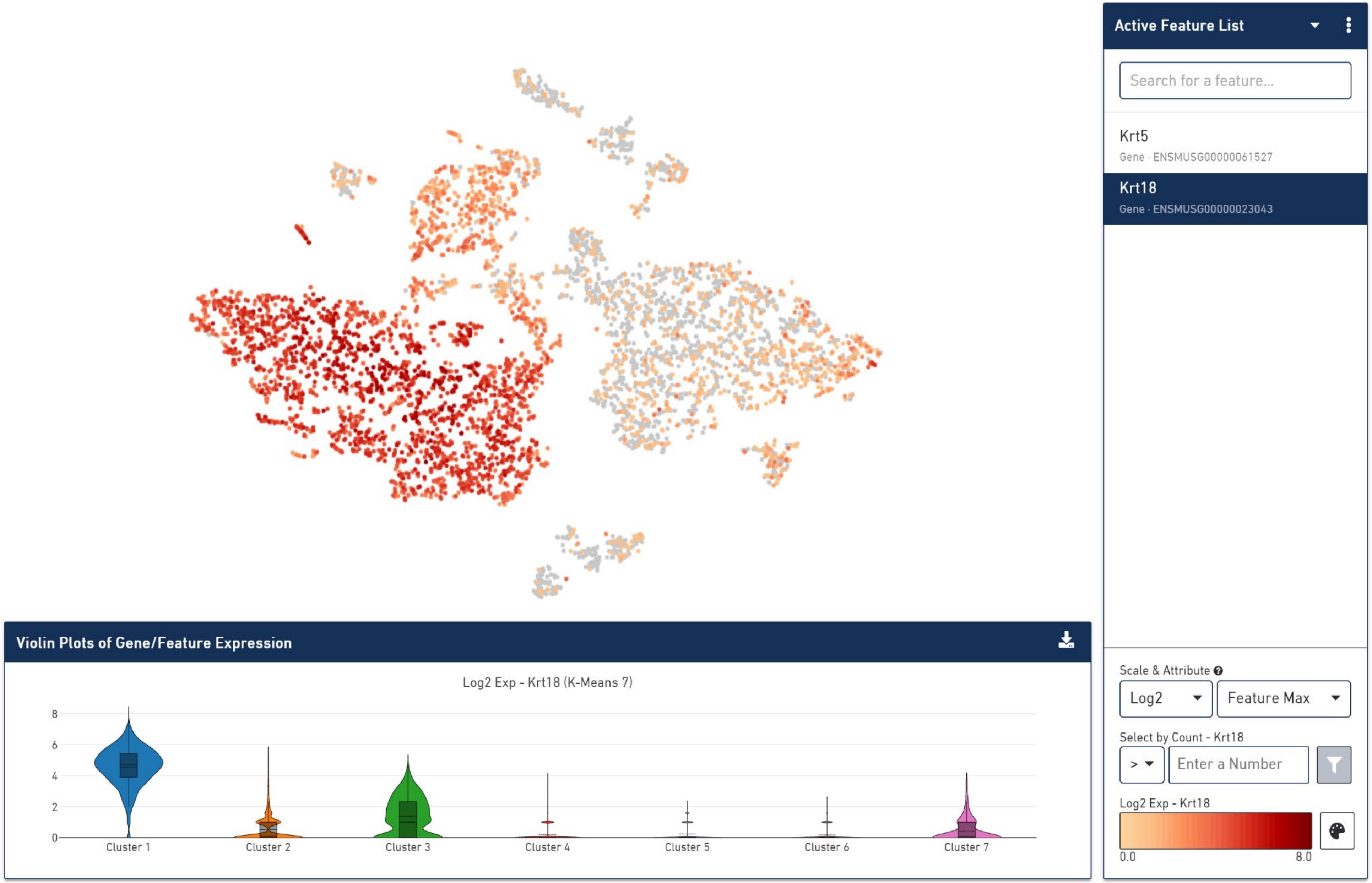

Stage: **Gestational**
Animal ID: **G1**
Clustering method: **tSNE**
Clustering: **Krt18**

Cluster 1 = **Krt18 high**
Cluster 2 = **Krt5 high**
Cluster 3 = **Krt18 low**
Clusters 4-7 = **other**

d.

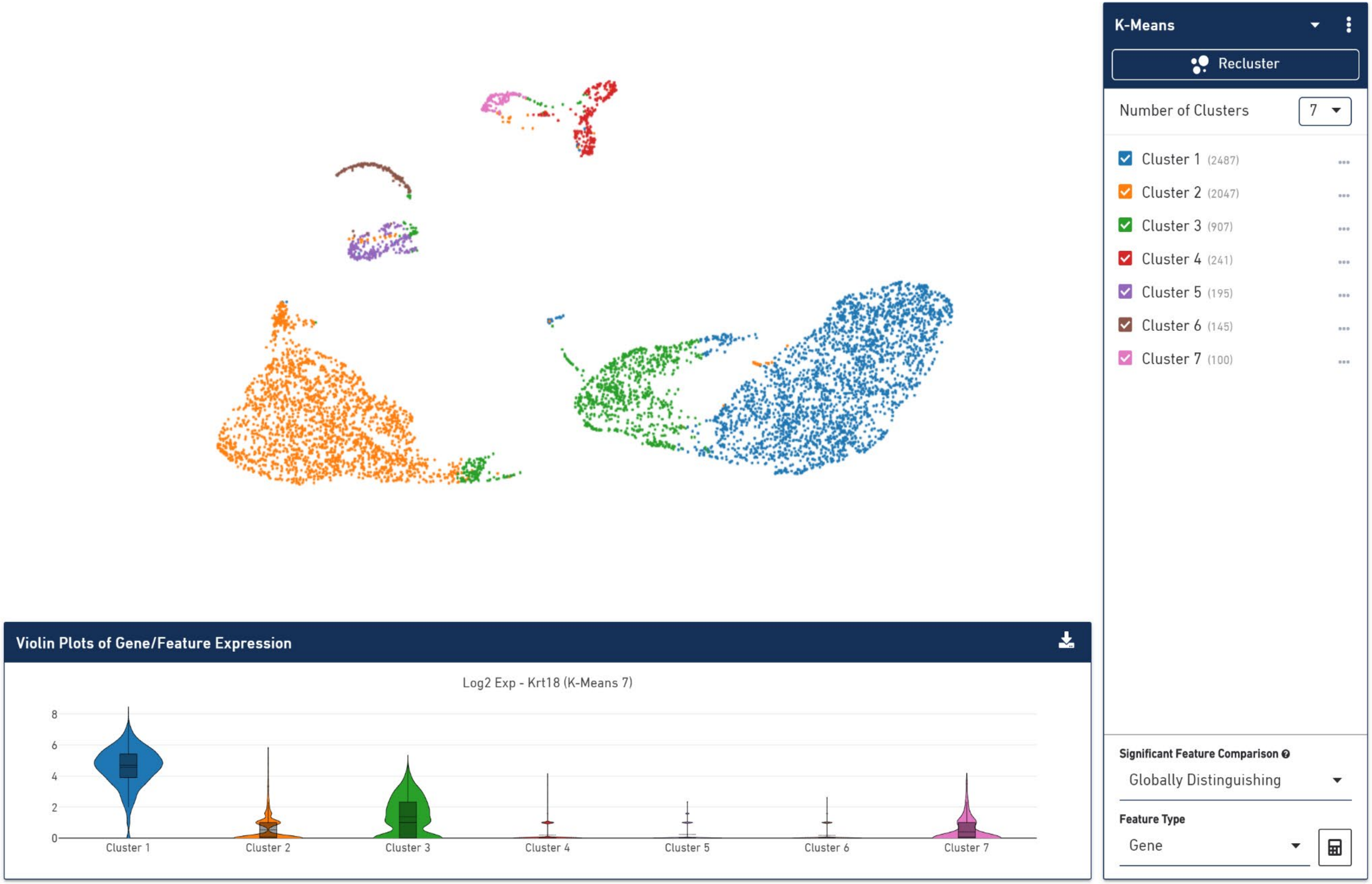

Stage: **Gestational**
Animal ID: **G1**
Clustering method: **UMAP**
Clustering : **K-means**

Cluster 1 = **Krt18 high**
Cluster 2 = **Krt5 high**
Cluster 3 = **Krt18 low**
Clusters 4-7 = **other**

e.

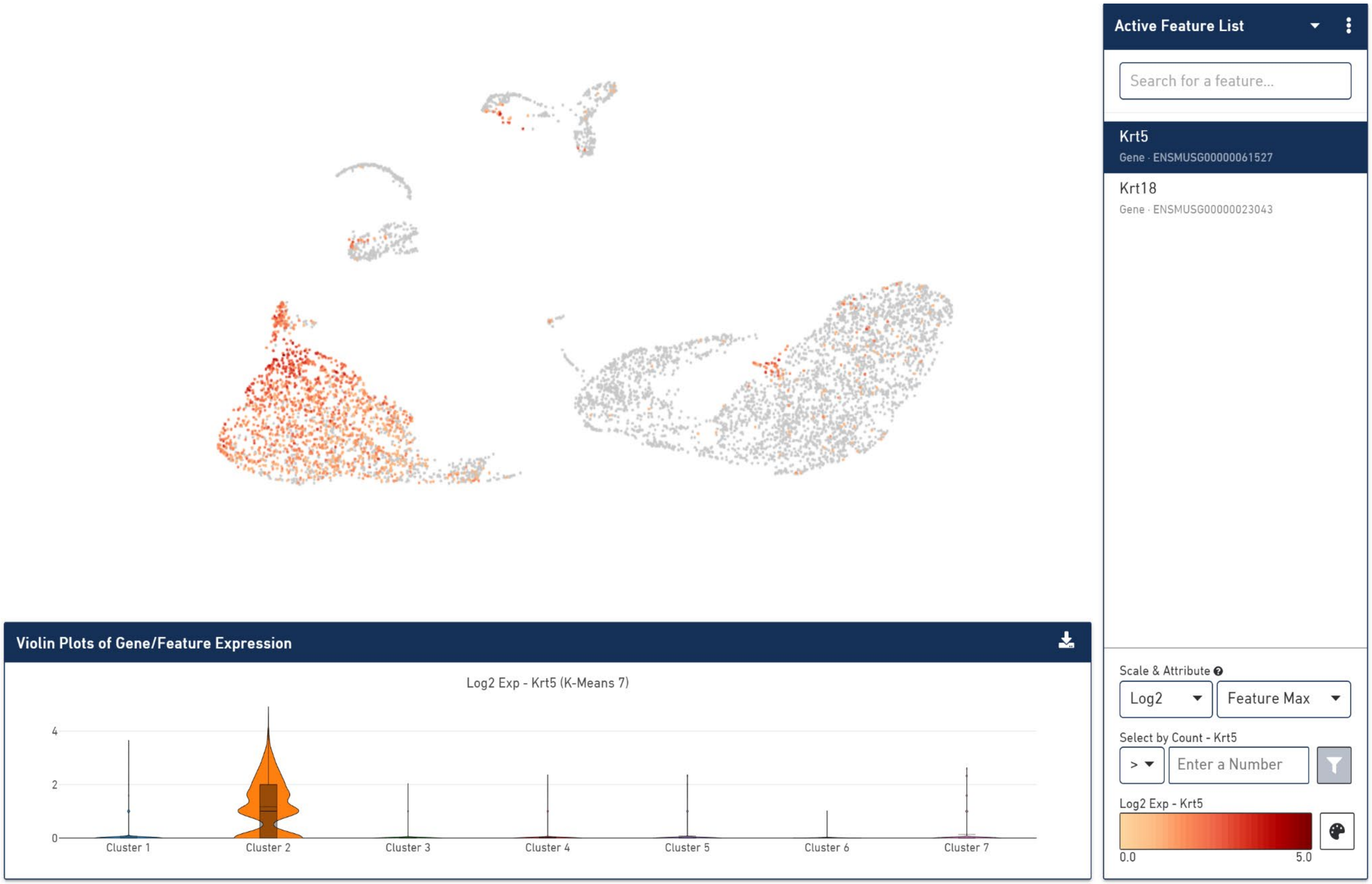

Stage: **Gestational**
Animal ID: **G1**
Clustering method: **UMAP**
Clustering: **Krt5**

Cluster 1 = **Krt18 high**
Cluster 2 = **Krt5 high**
Cluster 3 = **Krt18 low**
Clusters 4-7 = **other**

f.

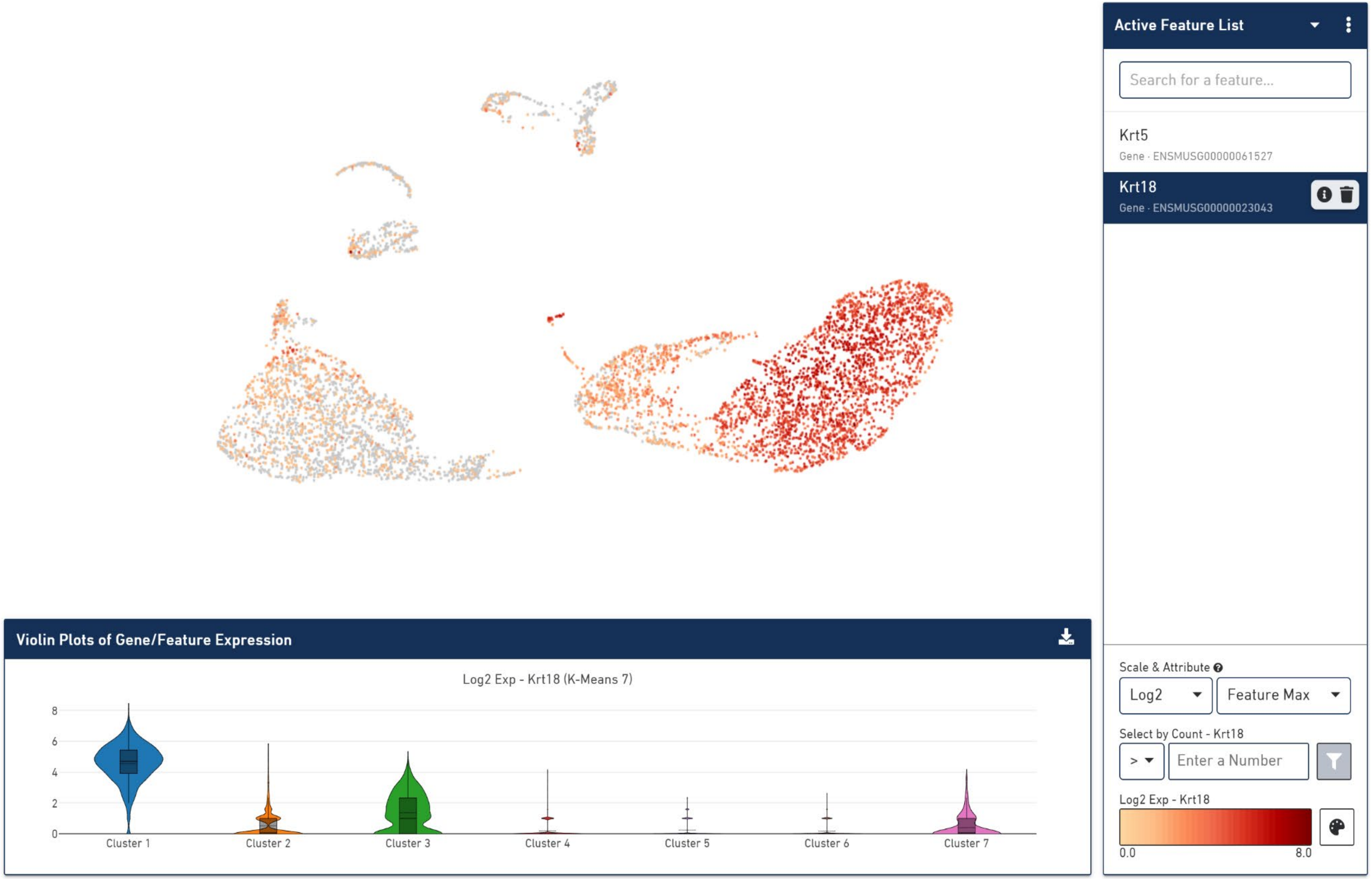

Stage: **Gestational**
Animal ID: **G1**
Clustering method: **UMAP**
Clustering: **Krt18**

Cluster 1 = **Krt18 high**
Cluster 2 = **Krt5 high**
Cluster 3 = **Krt18 low**
Clusters 4-7 = **other**

g.

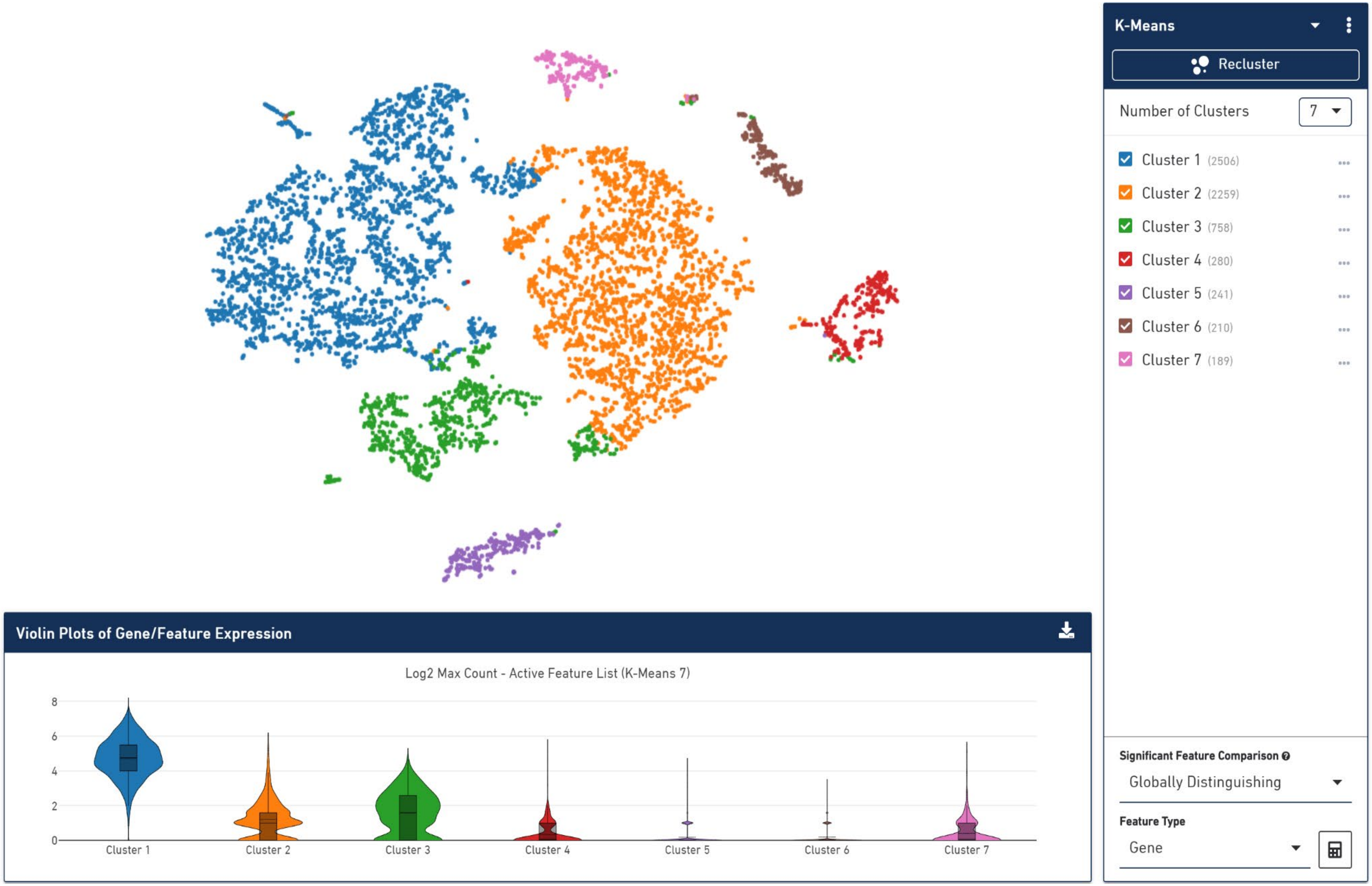

Stage: **Gestational**
Animal ID: **G2**
Clustering method: **tSNE**
Clustering: **K-means**

Cluster 1 = **Krt18 high**
Cluster 2 = **Krt5 high**
Cluster 3 = **Krt18 low**
Clusters 4-7 = **other**

h.

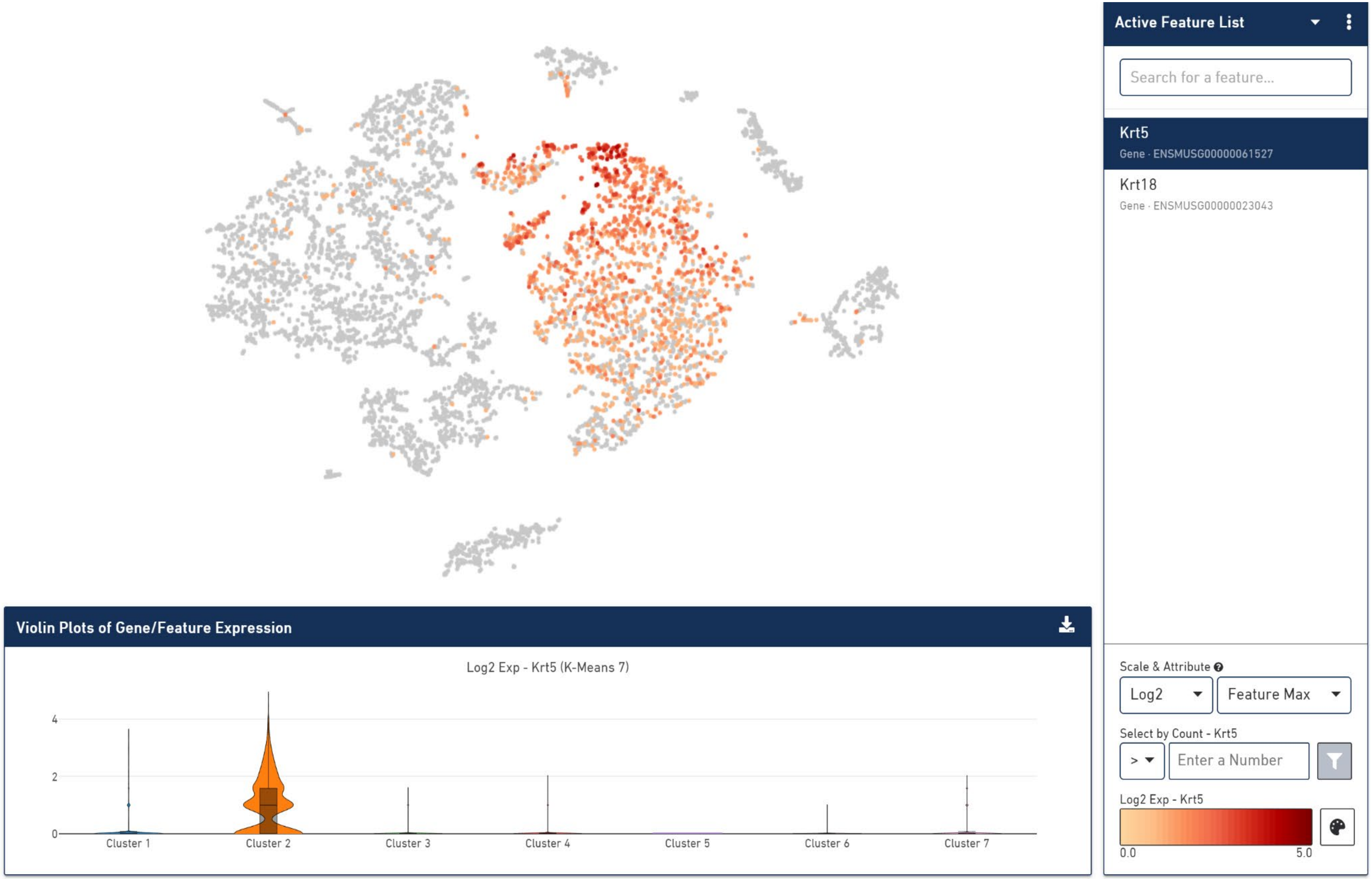

Stage: **Gestational**
Animal ID: **G2**
Clustering method: **tSNE**
Clustering: **Krt5**

Cluster 1 = **Krt18 high**
Cluster 2 = **Krt5 high**
Cluster 3 = **Krt18 low**
Clusters 4-7 = **other**

i.

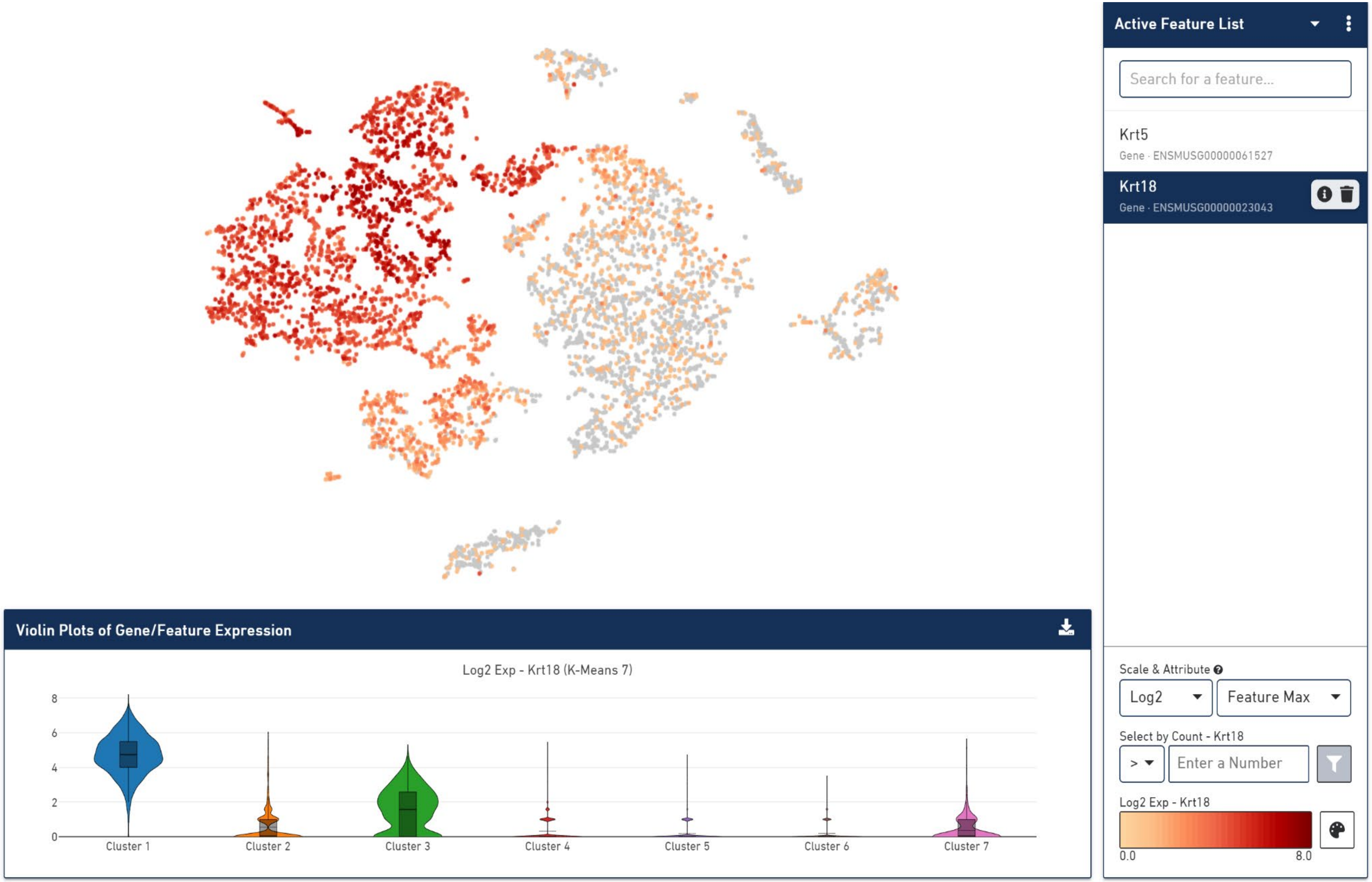

Stage: **Gestational**
Animal ID: **G2**
Clustering method: **tSNE**
Clustering: **Krt18**

Cluster 1 = **Krt18 high**
Cluster 2 = **Krt5 high**
Cluster 3 = **Krt18 low**
Clusters 4-7 = **other**

j.

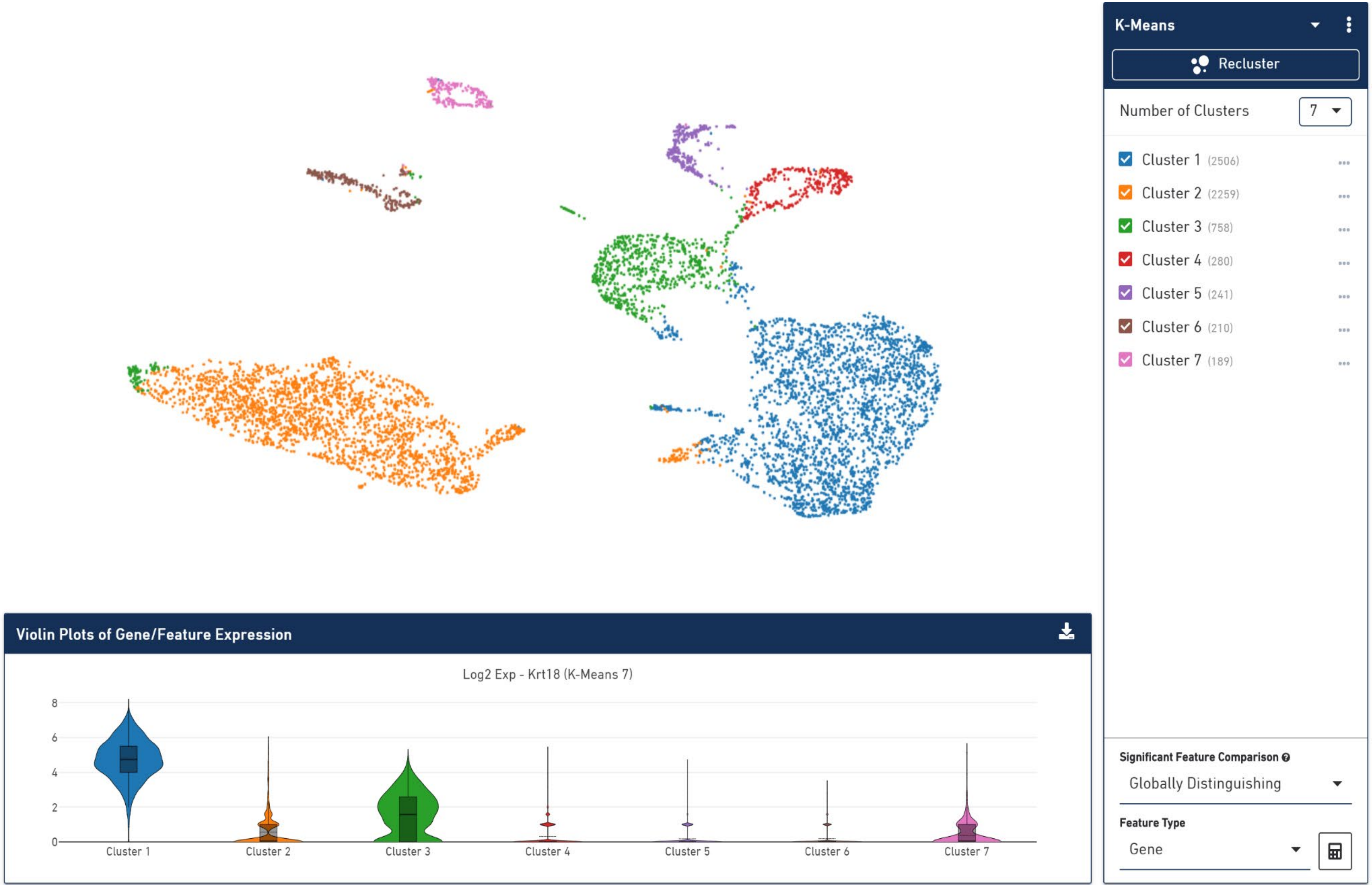

Stage: **Gestational**
Animal ID: **G2**
Clustering method: **UMAP**
Clustering: **K-means**

Cluster 1 = **Krt18 high**
Cluster 2 = **Krt5 high**
Cluster 3 = **Krt18 low**
Clusters 4-7 = **other**

k.

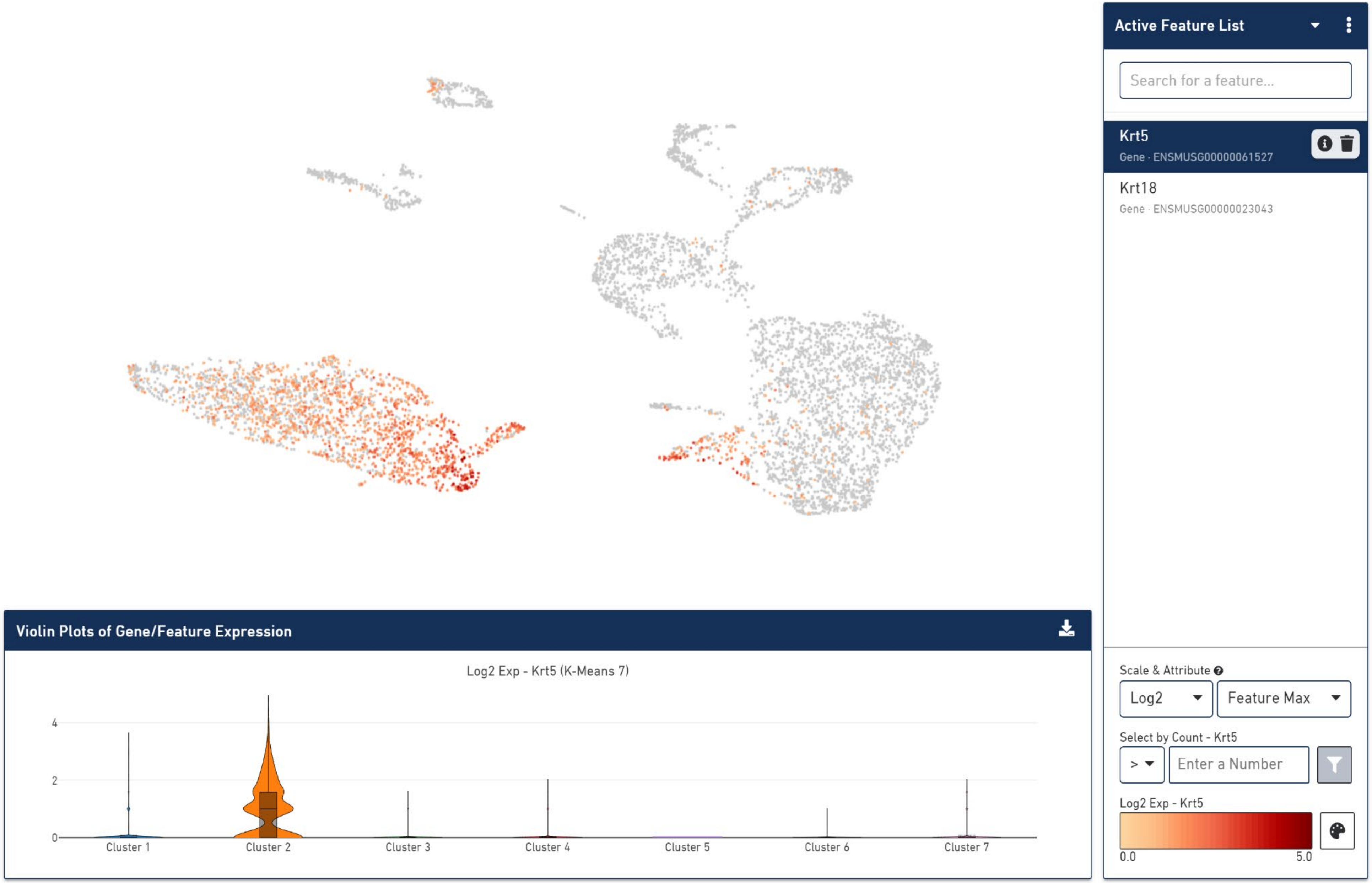

Stage: **Gestational**
Animal ID: **G2**
Clustering method: **UMAP**
Clustering: **Krt5**

Cluster 1 = **Krt18 high**
Cluster 2 = **Krt5 high**
Cluster 3 = **Krt18 low**
Clusters 4-7 = **other**

I.

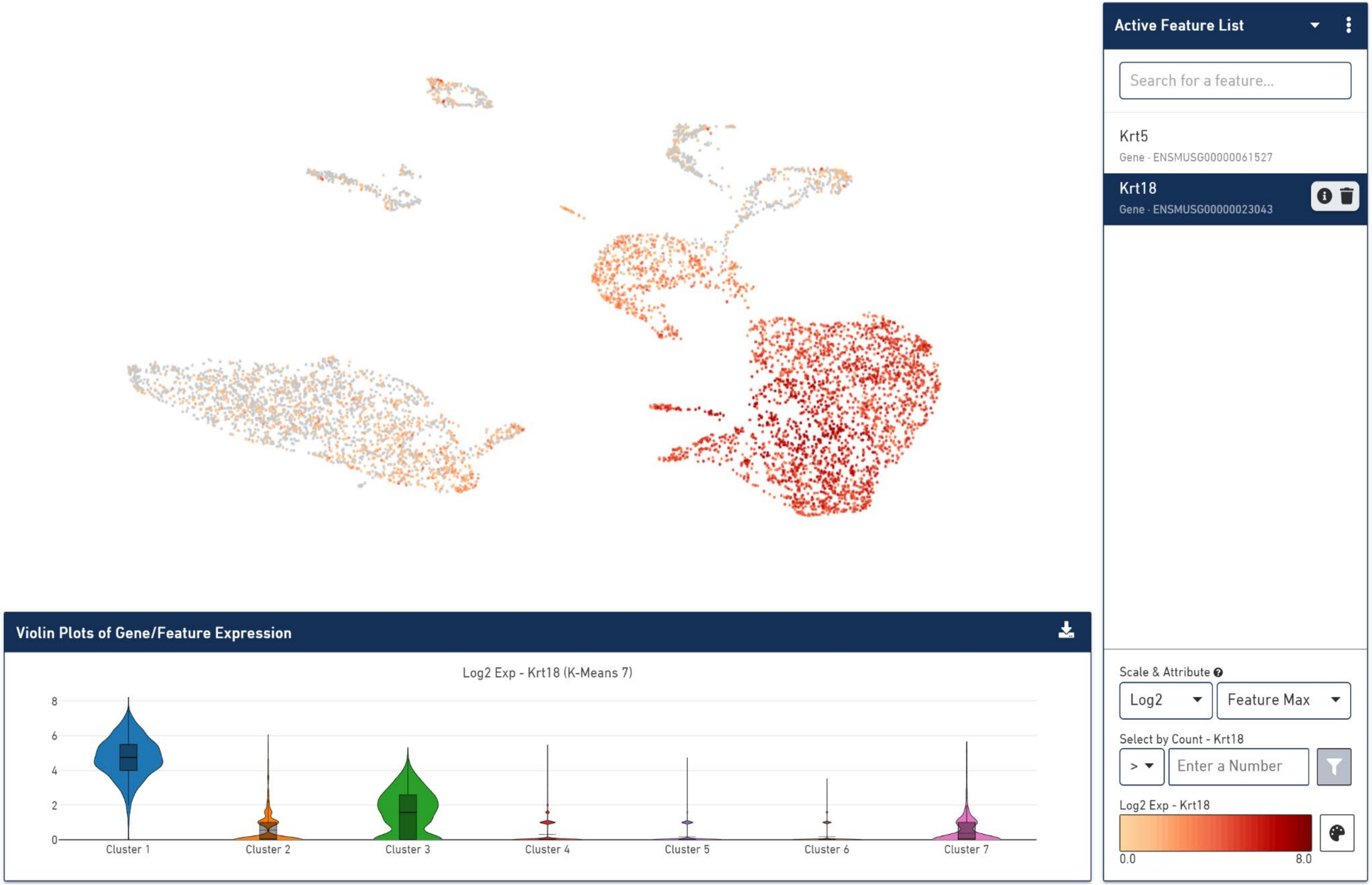

Stage: **Gestational**
Animal ID: **G2**
Clustering method: **UMAP**
Clustering: **Krt18**

Cluster 1 = **Krt18 high**
Cluster 2 = **Krt5 high**
Cluster 3 = **Krt18 low**
Clusters 4-7 = **other**

m.

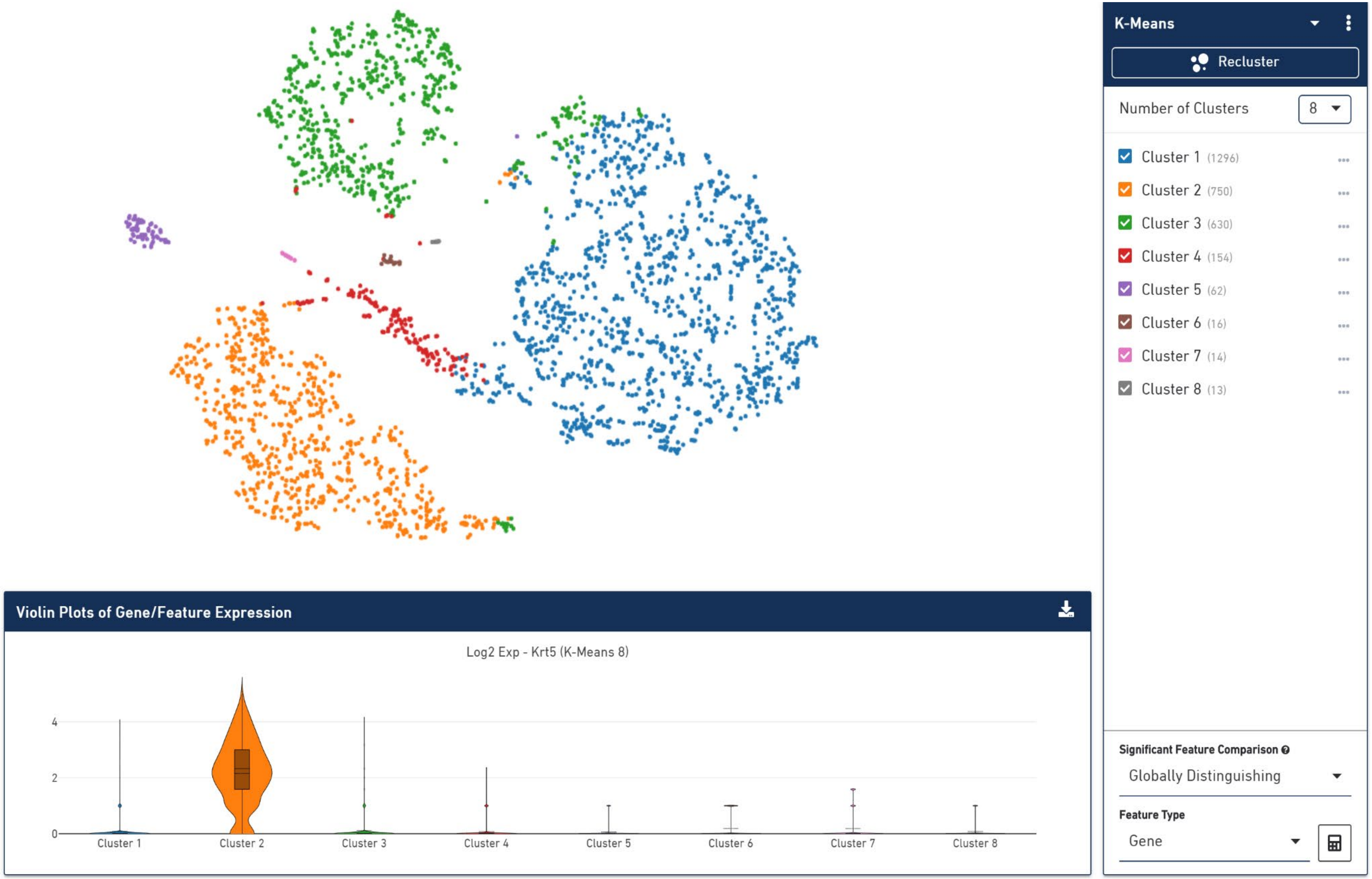

Stage: **Nulliparous**
Animal ID: **NP1**
Clustering method: **tSNE**
Clustering: **K-means**

Cluster 1,3 = **Krt18 high**
Cluster 2 = **Krt5 high**
Cluster 4,5 = **Krt18 low**
Cluster 6-8 = **other**

n.

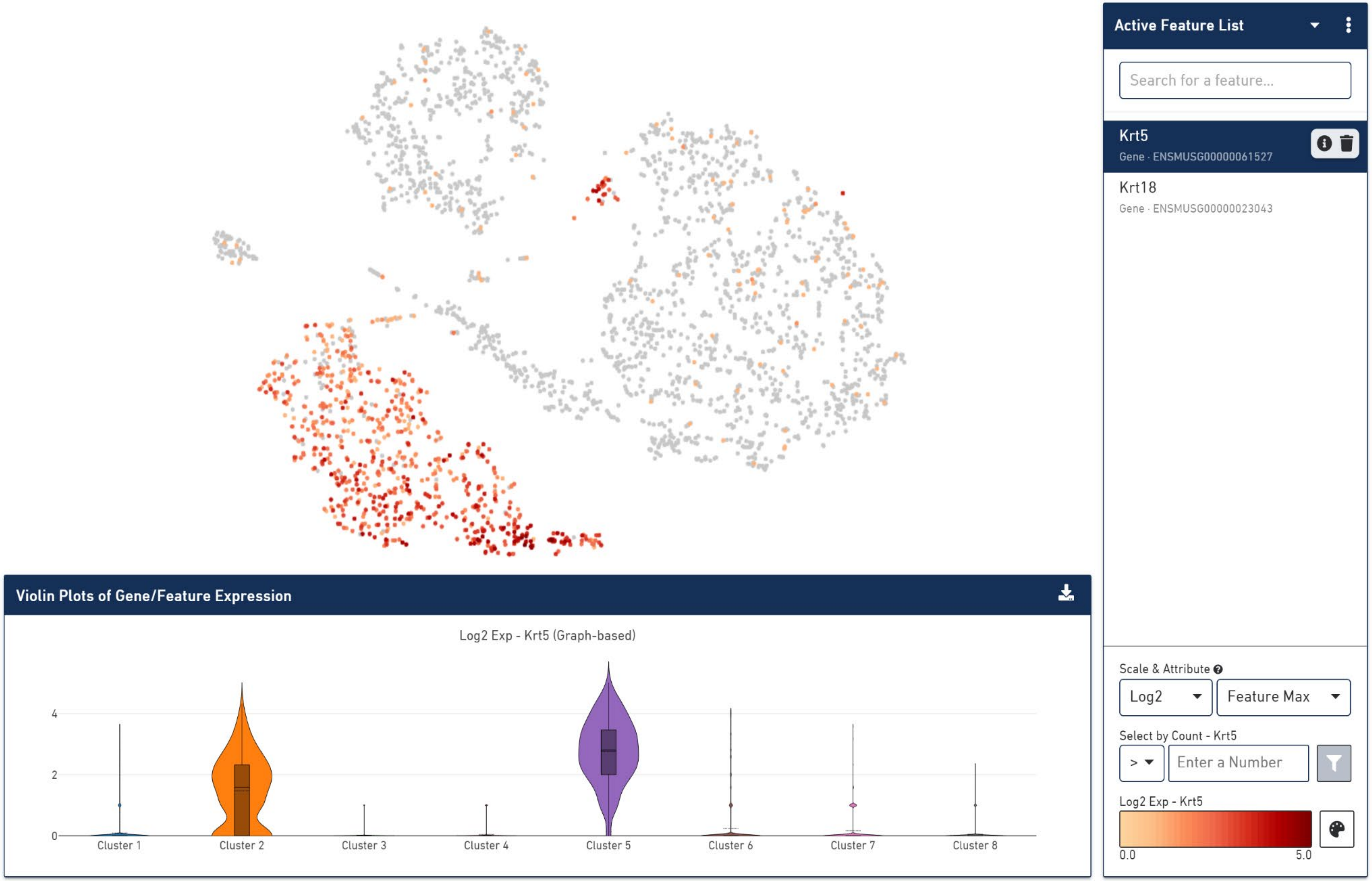

Stage: **Nulliparous**
Animal ID: **NP1**
Clustering method: **tSNE**
Clustering: **Krt5**

Cluster 1,3 = **Krt18 high**
Cluster 2 = **Krt5 high**
Cluster 4,5 = **Krt18 low**
Cluster 6-8 = **other**

o.

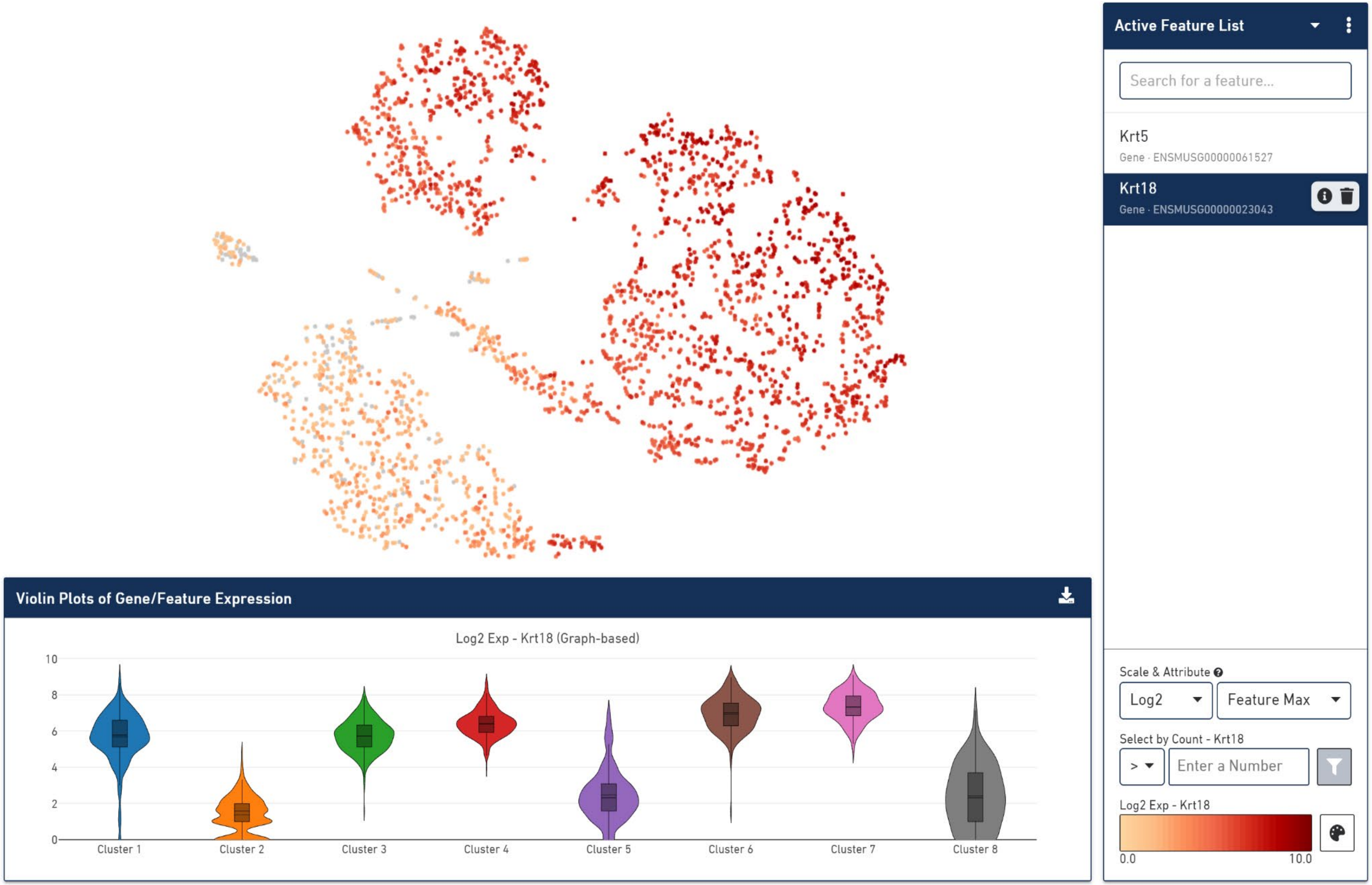

Stage: **Nulliparous**
Animal ID: **NP1**
Clustering method: **tSNE**
Clustering: **Krt18**

Cluster 1,3 = **Krt18 high**
Cluster 2 = **Krt5 high**
Cluster 4,5 = **Krt18 low**
Cluster 6-8 = **other**

p.

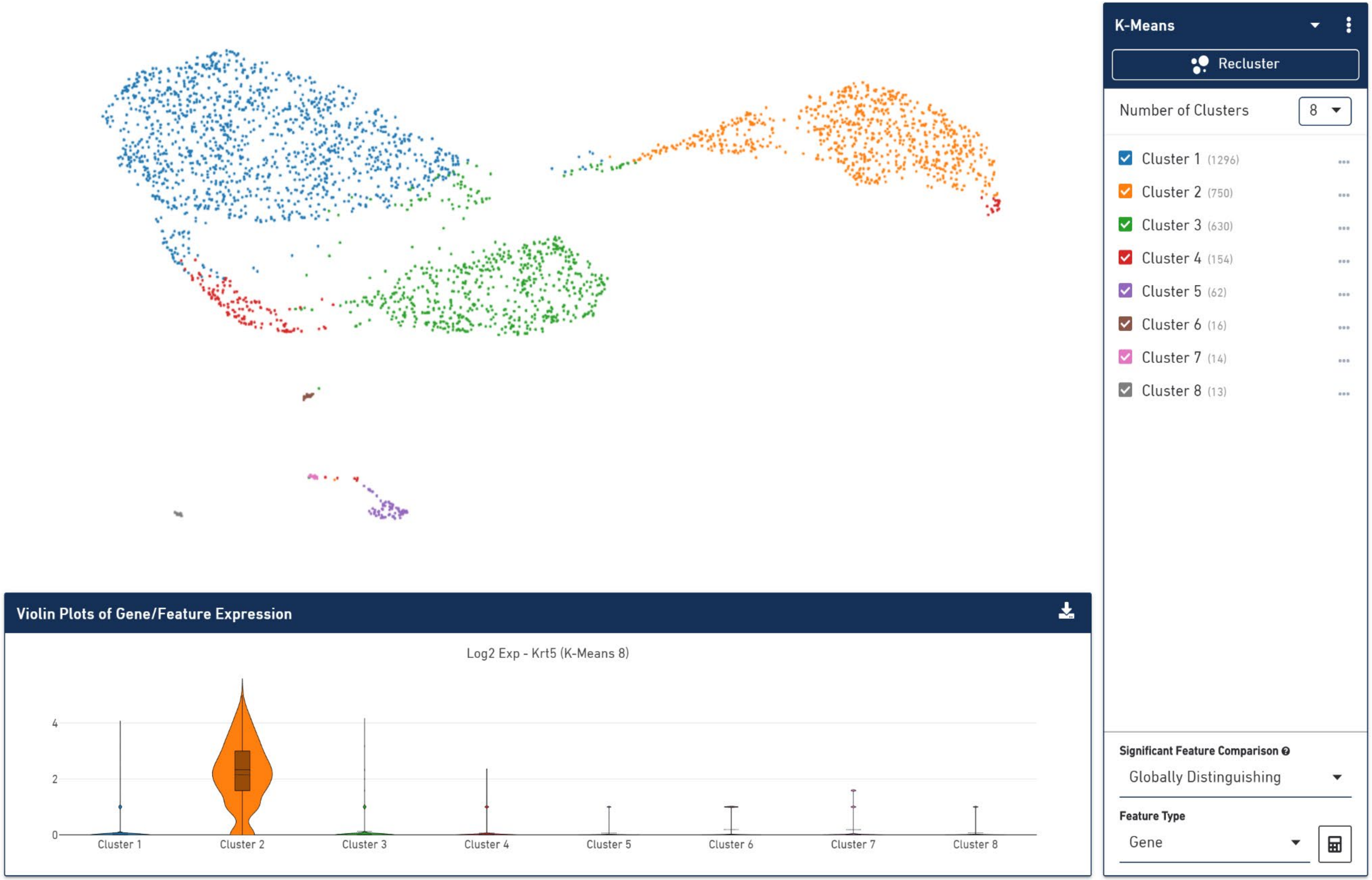

Stage: **Nulliparous**
Animal ID: **NP1**
Clustering method: **UMAP**
Clustering: **K-means**

Cluster 1,3 = **Krt18 high**
Cluster 2 = **Krt5 high**
Cluster 4,5 = **Krt18 low**
Cluster 6-8 = **other**

q.

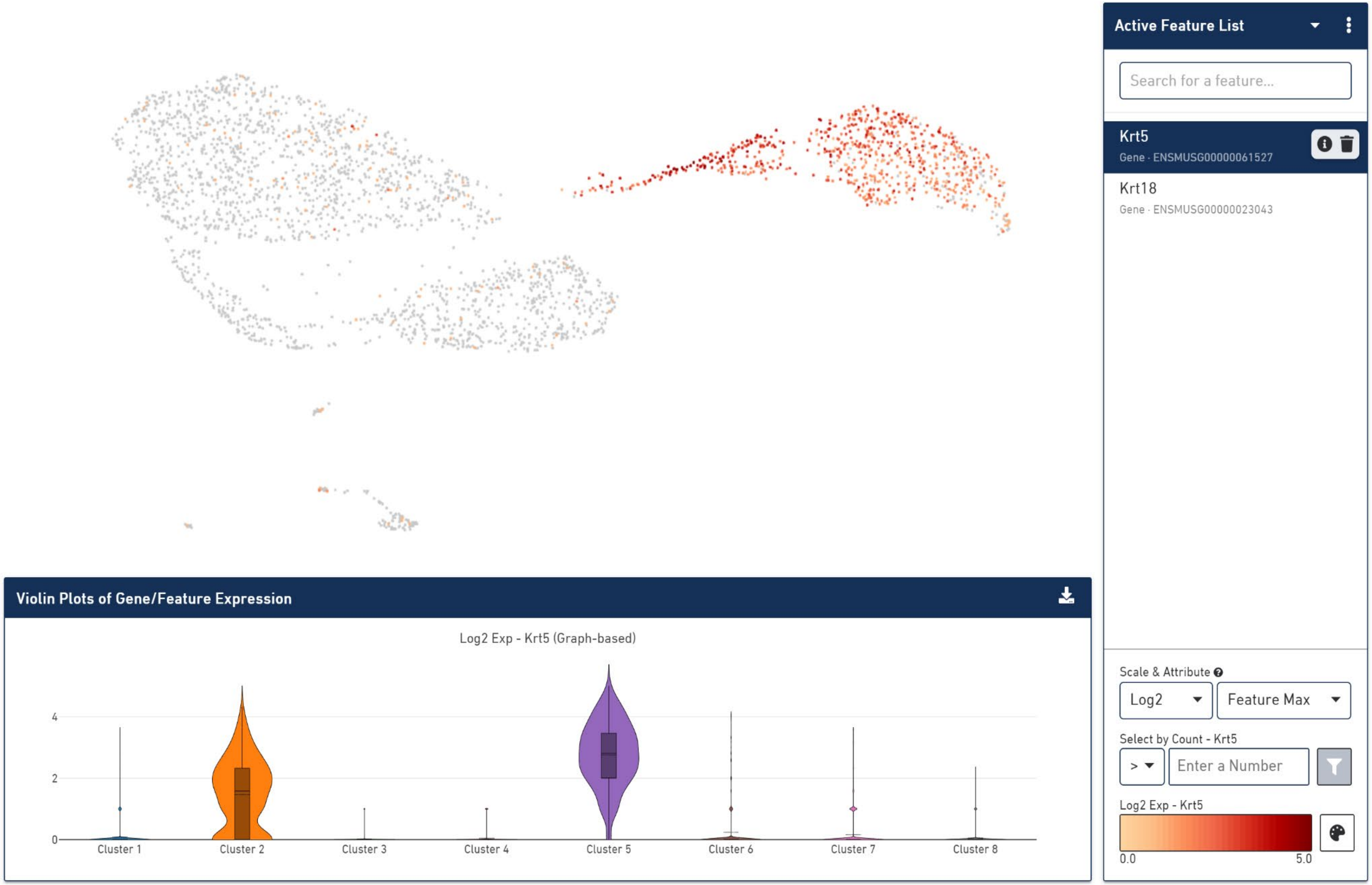

Stage: **Nulliparous**
Animal ID: **NP1**
Clustering method: **UMAP**
Clustering: **Krt5**

Cluster 1,3 = **Krt18 high**
Cluster 2 = **Krt5 high**
Cluster 4,5 = **Krt18 low**
Cluster 6-8 = **other**

r.

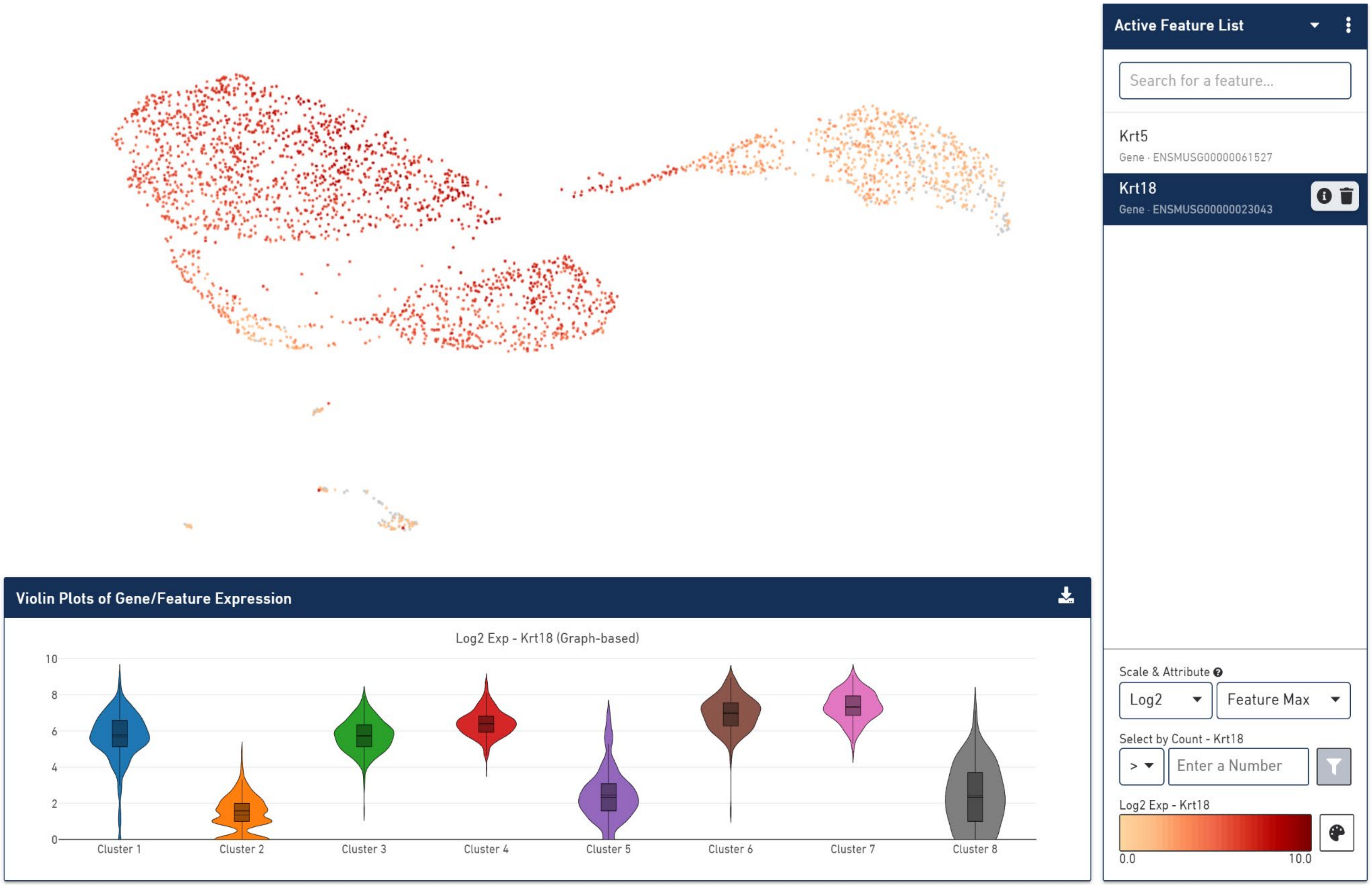

Stage: **Nulliparous**
Animal ID: **NP1**
Clustering method: **UMAP**
Clustering: **Krt18**

Cluster 1,3 = **Krt18 high**
Cluster 2 = **Krt5 high**
Cluster 4,5 = **Krt18 low**
Cluster 6-8 = **other**

s.

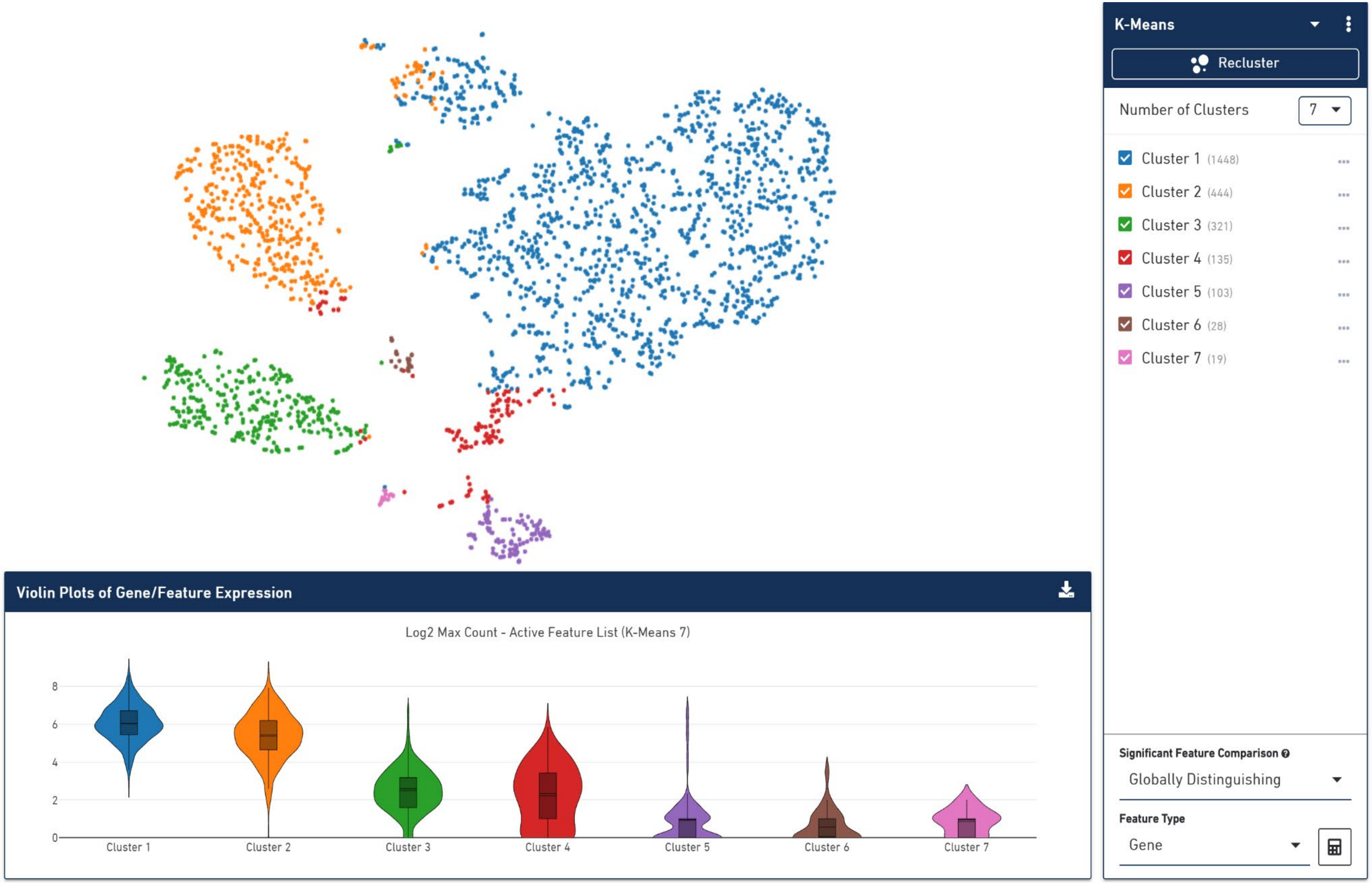

Stage: **Nulliparous**
Animal ID: **NP2**
Clustering method: **tSNE**
Clustering: **K-means**

Cluster 1,2 = **Krt18 high**
Cluster 3= **Krt5 high**
Cluster 4,5 = **Krt18 low**
Cluster 6-8 = **other**

t.

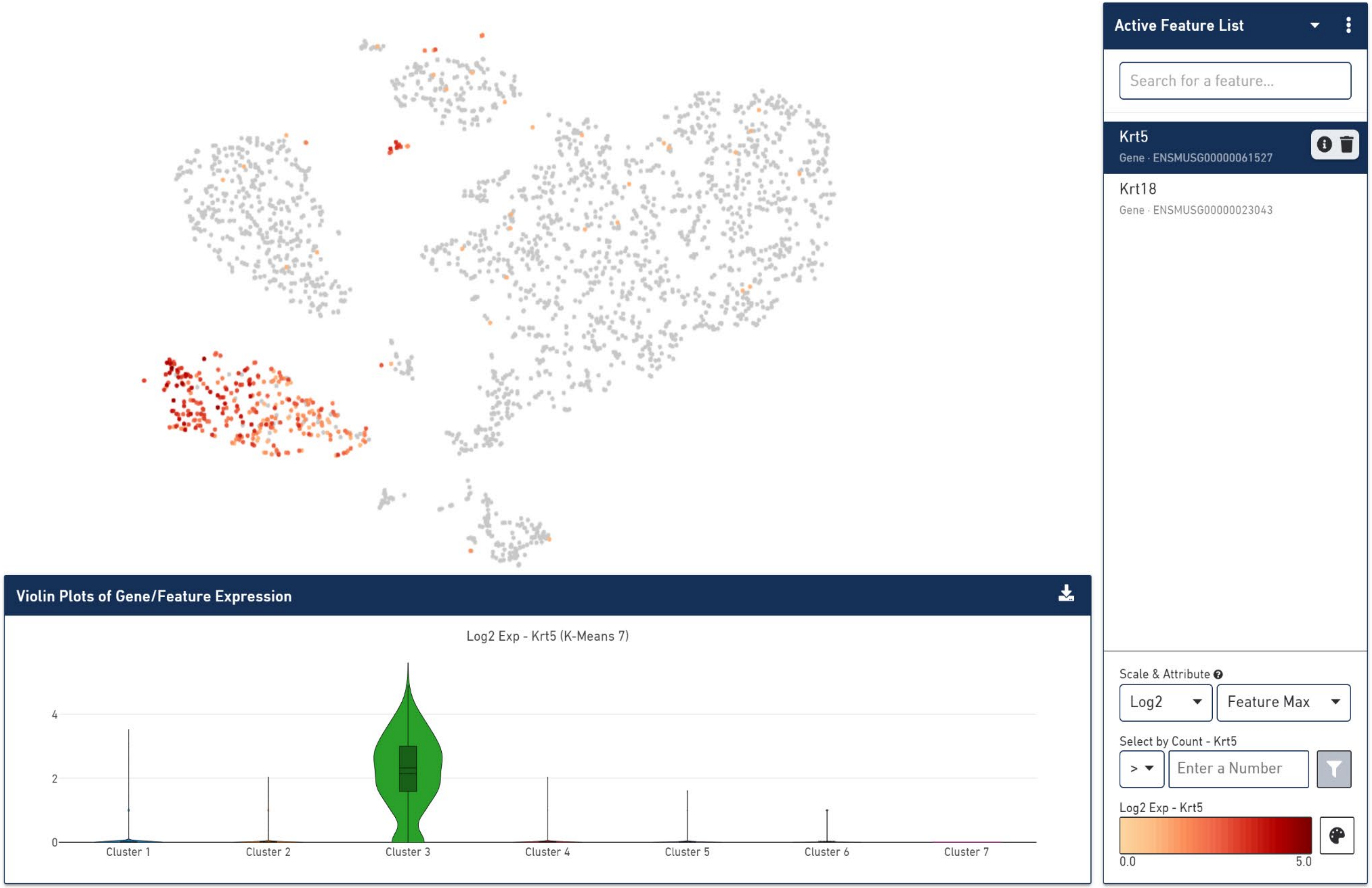

Stage: **Nulliparous**
Animal ID: **NP2**
Clustering method: **tSNE**
Clustering: **Krt5**

Cluster 1,2 = **Krt18 high**
Cluster 3= **Krt5 high**
Cluster 4,5 = **Krt18 low**
Cluster 6-8 = **other**

u.

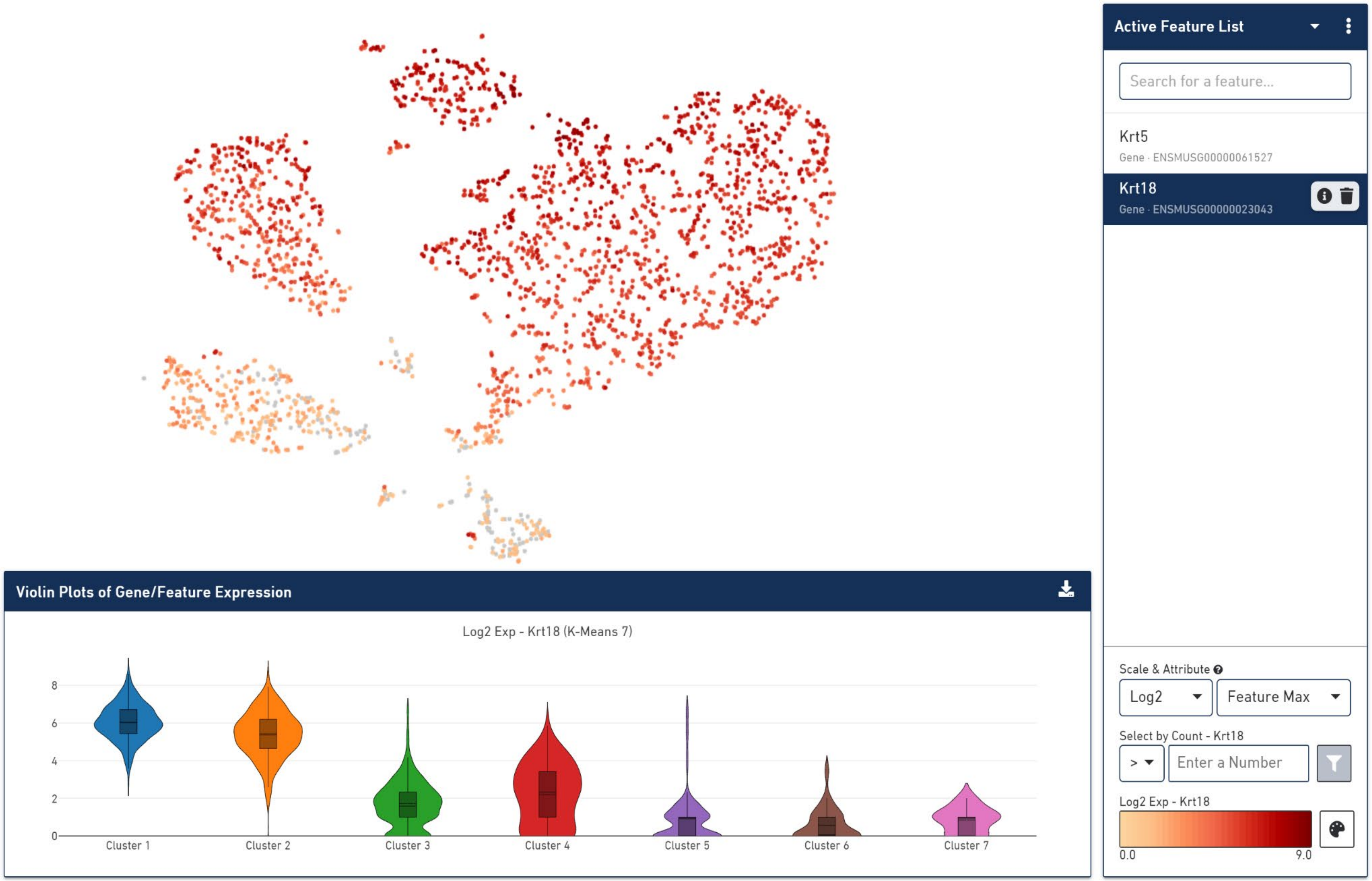

Stage: **Nulliparous**
Animal ID: **NP2**
Clustering method: **tSNE**
Clustering: **Krt18**

Cluster 1,2 = **Krt18 high**
Cluster 3= **Krt5 high**
Cluster 4,5 = **Krt18 low**
Cluster 6-8 = **other**

V.

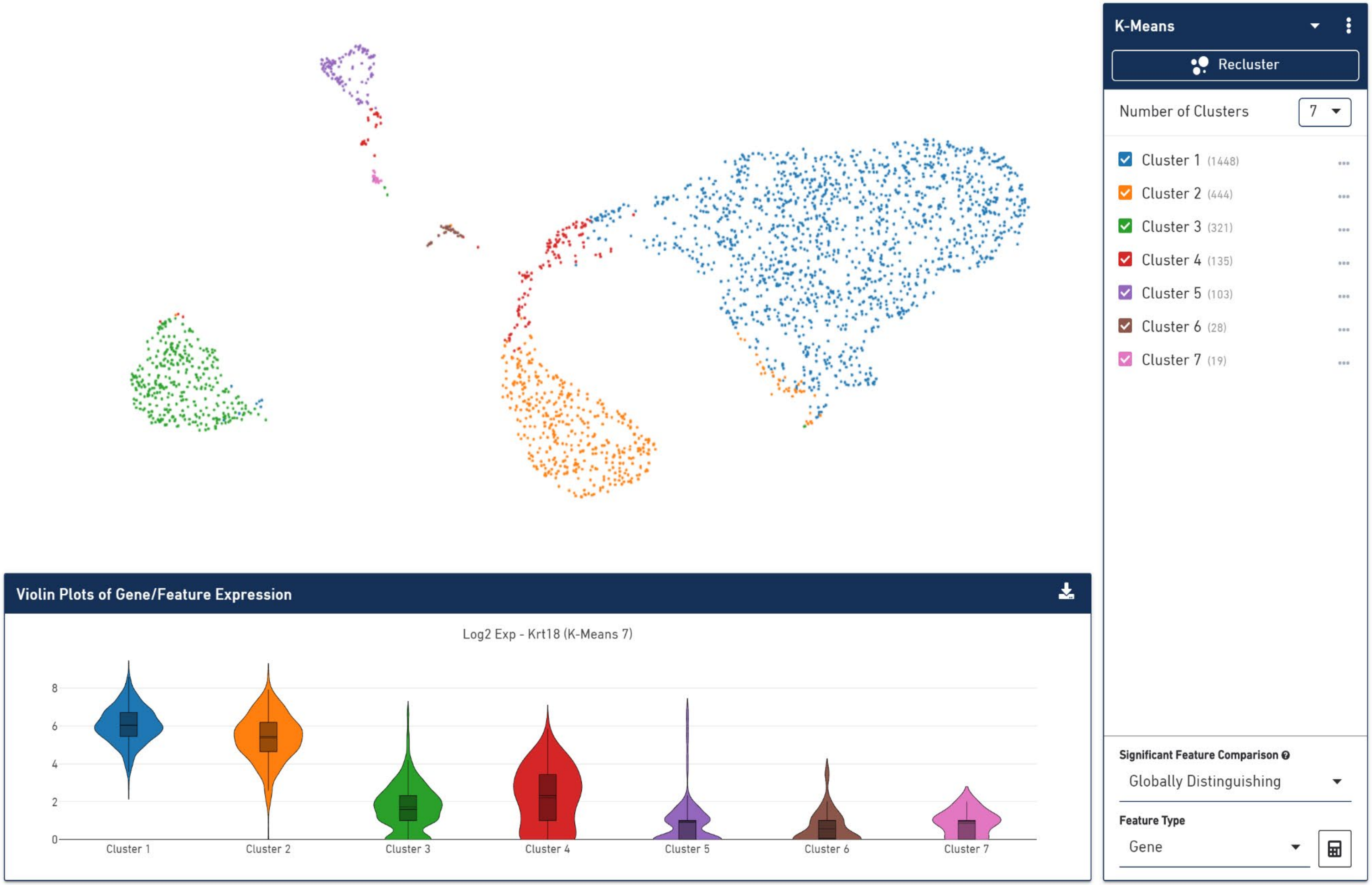

Stage: **Nulliparous**
Animal ID: **NP2**
Clustering method: **UMAP**
Clustering: **K-means**

Cluster 1,2 = **Krt18 high**
Cluster 3= **Krt5 high**
Cluster 4,5 = **Krt18 low**
Cluster 6-8 = **other**

w.

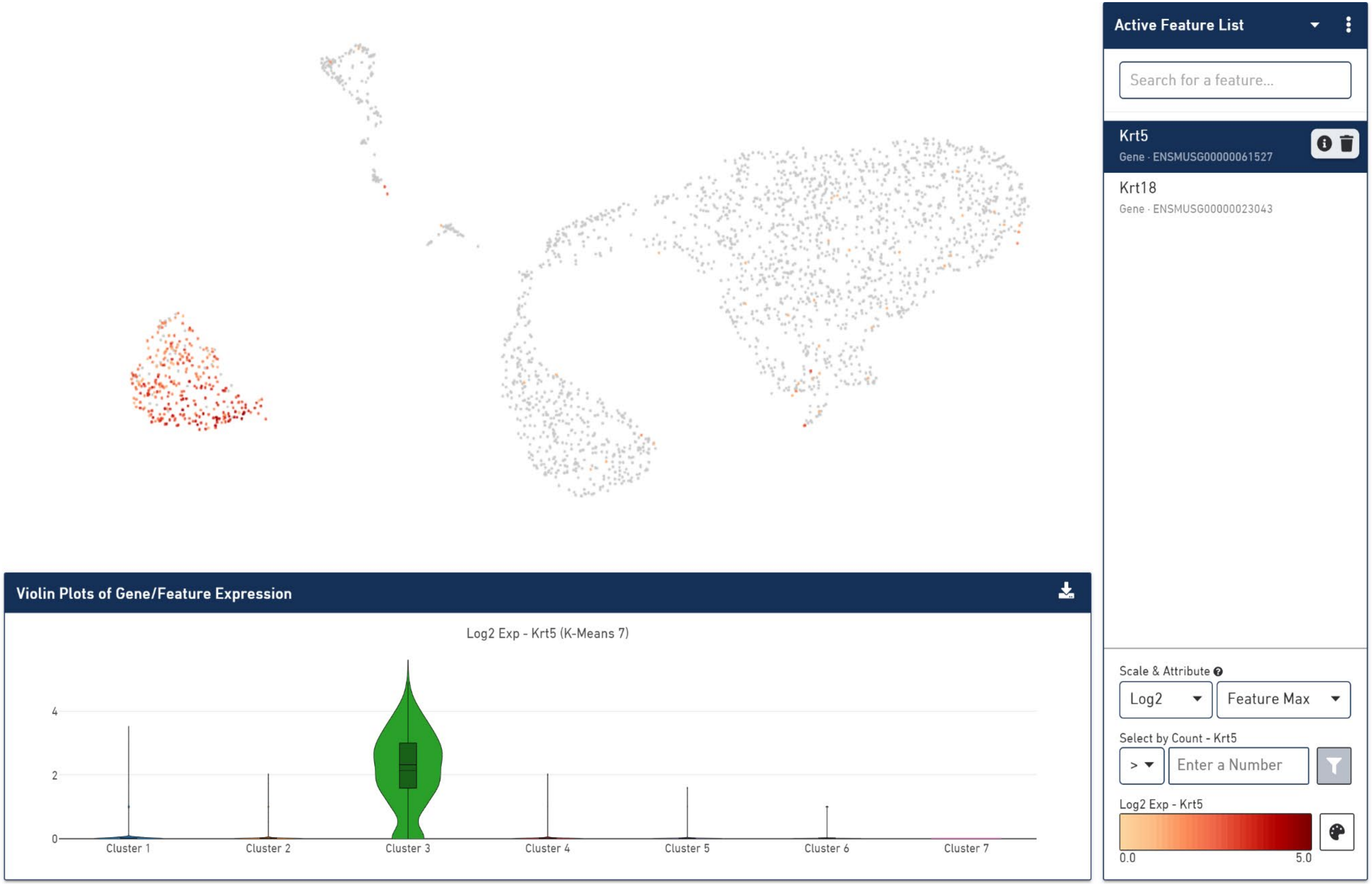

Stage: **Nulliparous**
Animal ID: **NP2**
Clustering method: **UMAP**
Clustering: **Krt5**

Cluster 1,2 = **Krt18 high**
Cluster 3= **Krt5 high**
Cluster 4,5 = **Krt18 low**
Cluster 6-8 = **other**

x.

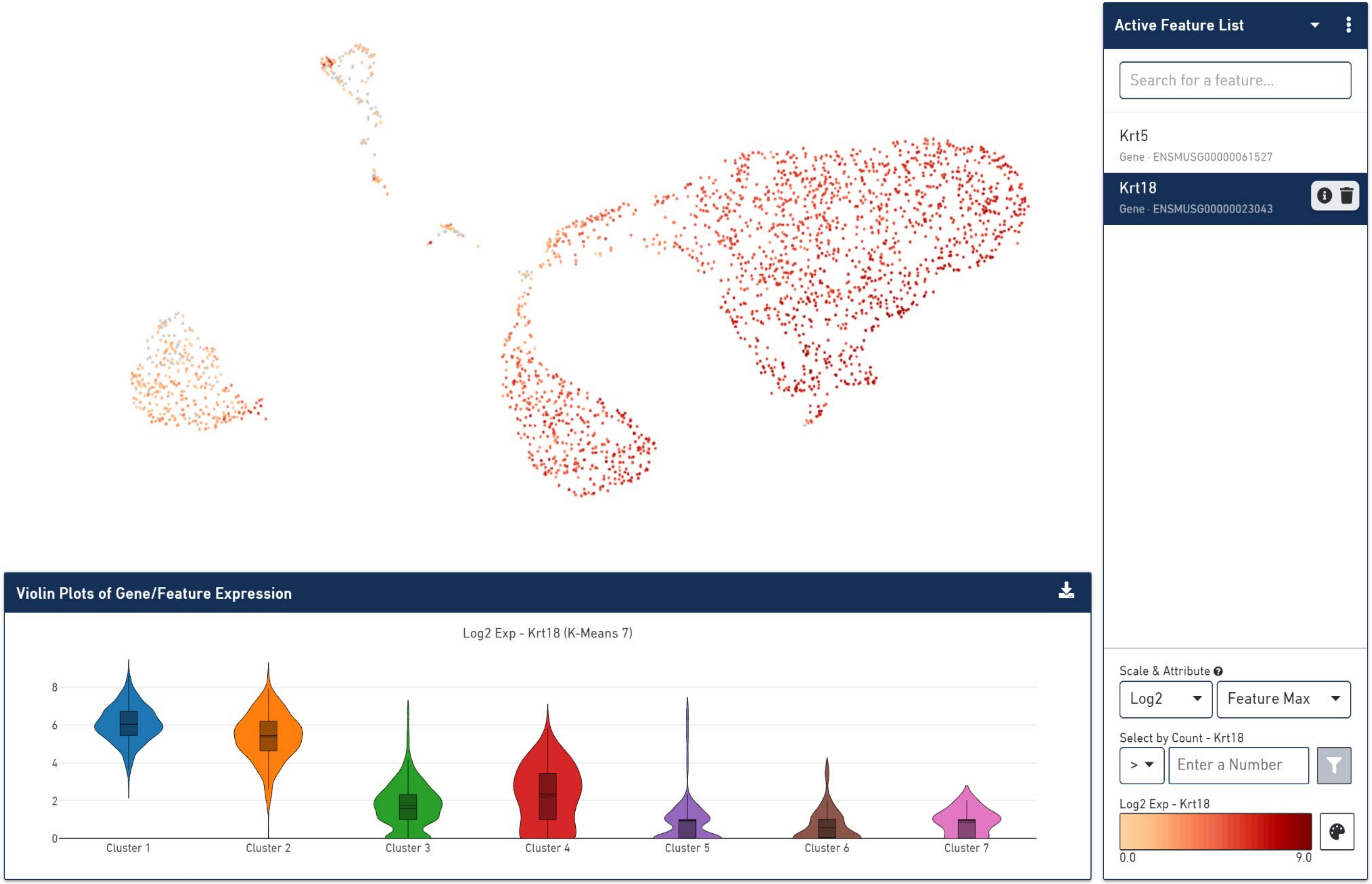

Stage: **Nulliparous**
Animal ID: **NP2**
Clustering method: **UMAP**
Clustering: **Krt18**

Cluster 1,2 = **Krt18 high**
Cluster 3= **Krt5 high**
Cluster 4,5 = **Krt18 low**
Cluster 6-8 = **other**
